# Filament-free memristors for computing

**DOI:** 10.1186/s40580-023-00407-0

**Published:** 2023-12-19

**Authors:** Sanghyeon Choi, Taehwan Moon, Gunuk Wang, J. Joshua Yang

**Affiliations:** 1 Department of Electrical and Computer Engineering, University of Southern California, Los Angeles, CA 90089 USA; 2 KU-KIST Graduate School of Converging Science and Technology, Korea University, 145 Anam-ro, Seongbuk-gu, Seoul, 02841 Republic of Korea; 3 Present Address: Department of Electrical and Computer Engineering, University of California, Santa Barbara, CA 93106, USA; 4 Department of Integrative Energy Engineering, Korea University, 145 Anam-ro, Seongbuk-gu, Seoul, 02841 Republic of Korea; 5 Center for Neuromorphic Engineering, Korea Institute of Science and Technology, Seoul, 02792 Republic of Korea

## Abstract

Memristors have attracted increasing attention due to their tremendous potential to accelerate data-centric computing systems. The dynamic reconfiguration of memristive devices in response to external electrical stimuli can provide highly desirable novel functionalities for computing applications when compared with conventional complementary-metal–oxide–semiconductor (CMOS)-based devices. Those most intensively studied and extensively reviewed memristors in the literature so far have been filamentary type memristors, which typically exhibit a relatively large variability from device to device and from switching cycle to cycle. On the other hand, filament-free switching memristors have shown a better uniformity and attractive dynamical properties, which can enable a variety of new computing paradigms but have rarely been reviewed. In this article, a wide range of filament-free switching memristors and their corresponding computing applications are reviewed. Various junction structures, switching properties, and switching principles of filament-free memristors are surveyed and discussed. Furthermore, we introduce recent advances in different computing schemes and their demonstrations based on non-filamentary memristors. This Review aims to present valuable insights and guidelines regarding the key computational primitives and implementations enabled by these filament-free switching memristors.

## Introduction

The last few decades have witnessed a brilliant improvement in computing technology, offering several critical benefits such as automation, effectiveness, analysis, and accuracy. One of the primary driving forces to the progress is the downscaling of electronic devices [1, 2]. The shrinkage approach could achieve the goal of reducing various device characteristics altogether, such as capacitance, voltage, current, transition time, and energy consumption [2–4]. This enabled a circuit to operate at a higher frequency with the same consumed energy, leading to an exponential increase of computing performance per Joule (referred to as Dennard scaling) [2, 4]. However, the breakdown of the Dennard scaling has appeared since around 2006 because the increase in the clock frequency at the same energy envelop started to saturate due to several fundamental limits [5, 6]; for example, as the device became dramatically shrunk, the leakage current induced by the tunneling effect has hampered the energy scaling [7, 8]. An alternative way to circumvent the end of the Dennard scaling era was the architectural shift from single core to multicore design by integrating multiple cores on a single chip [8, 9]. This solution has allowed each core to operate at a lower frequency and share various on-chip resources, thereby increasing the energy efficiency [9]. Nevertheless, the overall circuit power still remains excessive because of the number of operations increased by multiple cores, which makes it hard to concurrently power on all transistors at the nominal operating voltage (referred to as dark silicon issue) [10]. As a result, other measures related to device and architecture need to be considered to efficiently handle a wide range of compute-intensive tasks such as artificial intelligence (AI) related workloads.

**Fig. 1:**
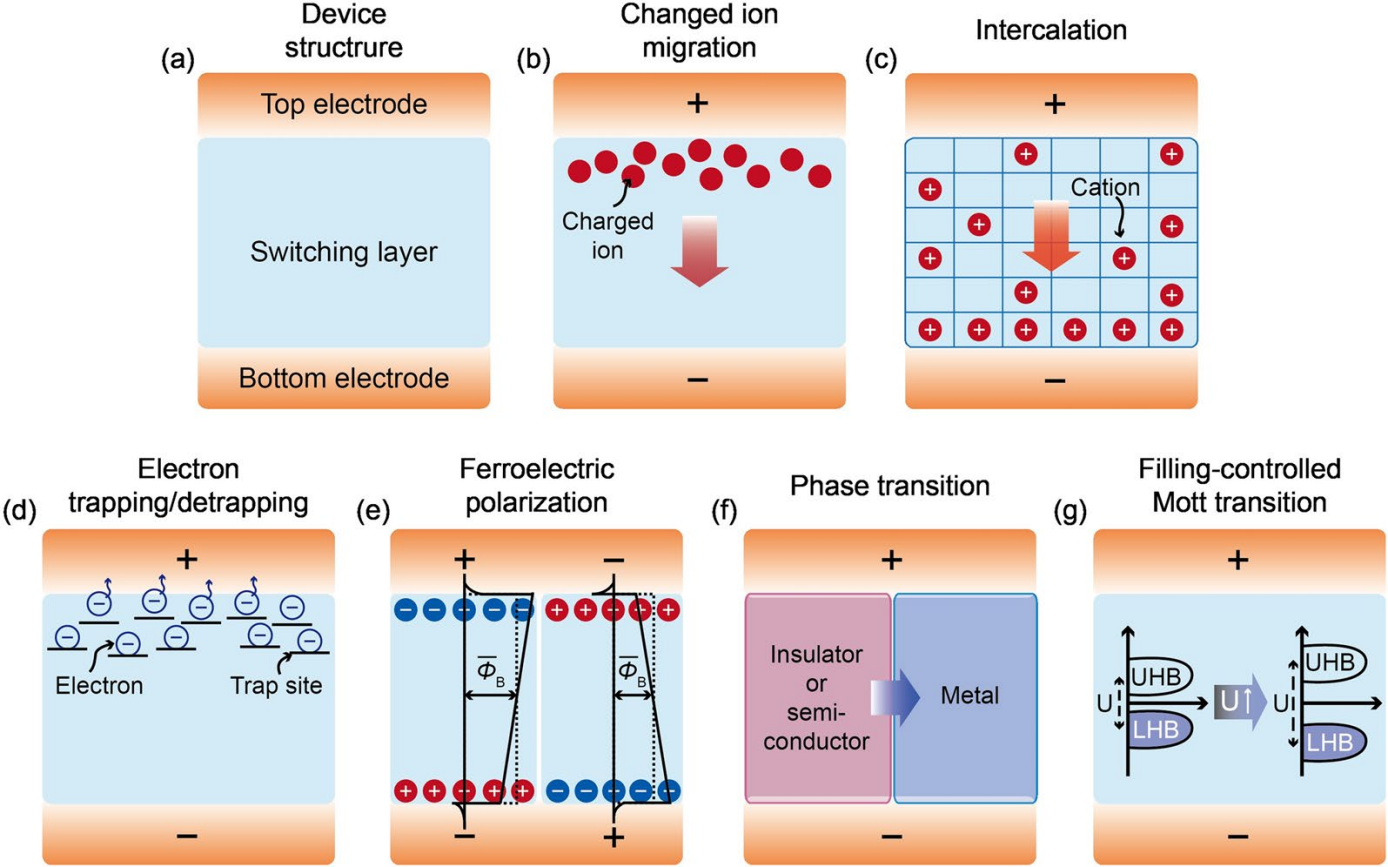
Filament-free switching memristor structures and their switching mechanisms. **a** A typical simple two-terminal memristor structure consisting of electrode/switching layer/electrode. **b**–**g** Illustrations of the switching principles for diverse types of filament-free switching memristors. **b** Ion migration. The switching is mainly governed by the electric field-driven migration of the ions. **c** Intercalation. The electric field-driven intercalation of ions into lattices is responsible for the main switching behavior. **d** Electron trapping/detrapping. The electronic (de)trapping processes at trap sites depending on the external electric field generates the switching events. **e** Ferroelectric polarization. The ferroelectric polarization switching results in the conductance switching. **f** Phase transition. A lower conductance phase (insulator or semiconductor) transforms into a higher conductance phase and vice versa. **g** Filling-controlled Mott transition. Mott–Hubbard gap increases or decreases corresponding to electron density in the transition metal orbitals

Under this circumstance, memristors and memristive computing architectures have widely attracted attentions due to their promising processing capability and energy efficiency [11–15]. The memristor, short for memory resistor, is an emerging two-terminal electronic device with a simple form of electrode/switching layer/electrode (Fig. 1a) that can alter its conductance state due to the dynamical reconfiguration of the switching layer induced by external electrical stimuli. As its name suggested, such conductance change can retain for a certain length of time with zero energy consumption. Moreover, multiple intermediate conductance states between ON (the low resistance state) and OFF (the high resistance state) state can be achieved via the use of appropriate electrical inputs. Furthermore, there have been experimental demonstrations of desirable switching properties such as nanoscale footprint (~ 2 nm) [16], low switching energy (< 1 pJ)[17], fast switching speed (< 100 ps) [18], and stable endurance (> 10^12^ cycles) [19]. Crossbar arrays with a high device density can be built with such devices to store and process data [11–15]. As a typical example, memristive crossbar array can readily perform vector–matrix multiplication and summation through the Ohm’s law and Kirchhoff’s law, respectively. The utilization of memristive computing architecture can facilitate the parallel and analog computation for a large amount of data with lower energy and latency cost than the conventional computing platforms. This is mainly because the burden of data shuttling between processor and memory units (referred to as von Neumann bottleneck) can be substantially alleviated [11–15, 20]. In addition, memristive computing may lead to a great reduction in the usage of analog-to-digital convertors (ADCs) or digital-to-analog convertors (DACs) when addressing various types of analog signals from sensory devices [21–23]. ADCs and DACs are notoriously known for their large energy, area and time consumptions [21, 24, 25]. Further, bio-inspired neuromorphic computing systems based on memristors are another promising research area because the dynamic ionic motion within the memristive switching layer closely mimics the similar processes in neurons, synapses and dendrites [14, 15]. This enables memristors to faithfully emulate such biological components. There have been demonstrations that essential biological functions such as integrate-and-fire dynamics, short- and long-term plasticity, paired-pulse facilitation/depression, and spiking-timing-dependent plasticity can be achieved with memristor based circuits [14, 15]. In this regard, it can be expected that a memristive neural network integrated with peripheral circuits and power sources may eventually be capable of brain-like computing functions and applications, such as pattern/speech recognition [14, 15, 20, 26], classification [27–29], and future prediction [23, 30–32]. Nevertheless, current memristive computing systems generally suffer from several undesired characteristics. Particularly, intrinsic stochasticity and non-uniformity in the switching events can impede the well-defined logical operation and necessitate repetitive correction processes [33–35], leading to the degradation of computing performance in terms of accuracy, energy, and time. Thus, the ability to interpret, engineer, and utilize memristors is a prerequisite to bringing the memristive computing systems into reality.

Memristors can be broadly classified into filament and filament-free types depending on whether the switching process occurs within a few localized conduction channels or uniformly across the entire device area [11, 36–39]. To date, filamentary switching memristors have been the most common ones existing in the literature because the filament offers an effective solution to overcome the speed-retention dilemma and achieve both high switching speed and long retention simultaneously within the same device [11, 36, 38]. This is because filament is so thin that it can be heated up quickly with even a small amount of current (but high current density) during the switching process. Once heated up, mobility of ions increases exponentially, resulting in a fast switching speed. It is also because filament is so thin that it can be quickly cooled down at the end of the applied switching stimuli. Once cooled down, the mobility of ions decreases exponentially and ions migrated during switching process are ‘frozen’ in their new positions, leading to a great retention. Unfortunately, the switching filaments also bring about some challenges including the need of electroforming process, relatively non-uniform switching process, Joule heating issue, and lower device yield. To address these obstacles, several potential solutions have been proposed including heterojunction structure [40], insertion of barrier layer [41], defect control [42, 43], operating scheme [44], and interface engineering [45]. Meanwhile, there have been increasing interests in the filament-free type of memristors because it promises a relatively small variability, a lower operating current, and a higher device yield without the initial forming process [37, 39]. These switching properties could be achieved by the uniform and homogeneous operating principles, attributed to the physical or chemical phenomena uniformly across the entire switching layer and interfacial contact. However, these mechanisms are prone to other issues, such as slow switching speed and short retention, which are undesirable for traditional memory applications. Nevertheless, most of filament-free switching memristors are capable of interesting dynamics that can enable some novel computing schemes. For instance, they can perform a non-linear transformation with respect to time-dependent inputs due to their nonlinear dynamic properties, which can be utilized to address high-order complex data (e.g., reservoir computing) [23, 30–32]. This makes a clear difference from many recent efforts on memristive computing applications that program a memristive crossbar array into desired conductance values and essentially just use the devices as static nonvolatile memory to perform inference processes [14, 15, 20, 26]. Therefore, understanding the non-filamentary memristors that are complementary to the filamentary devices is timely and crucial for realizing complex but efficient computing systems. Note that the general trends for the two types of memristors are summarized in Table 1, assuming an electrode/switching layer/electrode memristor with a thin film structure.

**Table 1:** General trends of representative characteristics according to the presence of switching filament

Type	Switching region	Initial forming process	feedback effect	Area dependency	Speed	Retention	Uniformity	Yield	Operating current
Filament-free	Whole area (or bulk)	No required	No	Significant	Slow	Short	Good	High	Low
Filament	Nanoscale filament (channel)	Required	Heat	Insignificant	Fast	Long	Poor	Low	High

Note that these trends are always not applicable to all the memristors. For example, filament-free memristors based on intercalation (Sect. 2.2) typically shows a relatively long retention time

In this Review, we provide an overview of the filament-free type of memristors and its computing applications. The Review is organized as follows. After introduction (Sect. 1), discussions on the comprehensive concepts and fundamental switching mechanisms of the filament-free switching memristors are presented along with some relevant recent efforts (Sects. 2 and 3). In Sect. 2, various types of operating principles are described: ion migration (Sect. 2.1), intercalation (Sect. 2.2), electron trapping/detrapping (Sect. 2.3), ferroelectric polarization (Sect. 2.4), phase transition (Sect. 2.5), and filling-controlled Mott transition (Sect. 2.6). Then, recent advances in these diverse types of filament-free switching memristors are sequentially introduced and discussed (Sects. 3.1, 3.2, 3.3, 3.4, 3.5, 3.6). Thereafter, we describe representative hardware implementations of computing applications based on the filament-free type of memristors from conceptualization to artificial synapses/neurons (Sect. 4.1 and 4.2), reservoir computing (Sect. 4.3), selector (Sect. 4.4), self-rectifying memristor (Sect. 4.5). Finally, a brief outlook and conclusions follow (Sect. 5). Until now, there have been a broad variety of Reviews regarding memristors and their computing application. For example, various topics and aspects have been reviewed with emphasis of memristive materials and devices [11, 36], theoretical progress [46–48], research methodologies [49], analysis scheme [50, 51], crossbar array [14, 52], circuit/architecture [53], and biological inspiration and algorithm [54, 55]. More specifically, many of the previous Reviews have covered filamentary switching memristors, their junction structures, their switching principles, and their hardware implementation of neuromorphic computing in recent years. In such reviews, the non-filamentary switching memristors and their future memory applications may also have been touched upon, but usually very briefly. Our Review here is dedicated to the filament-free type of memristors, highlighting their switching mechanisms and applications for diverse computing technologies that have become increasingly important.

## Operating principles of filament-free switching memristors

For the filamentary type of memristors, the migration of electrochemically active metallic ions (e.g., Ag, Cu) and defects (e.g., oxygen vacancies) induced by external electric stimuli mainly contributes to the growth and rupture of localized conductive pathways within the switching layer[11, 36, 38]. Because the switching event occurs locally, it can be expected that the ON current is almost independent of device area until the device area is reduced to the size of the switching filament. By contrast, the filament-free type of memristors is associated with the homogeneous conduction and insulation effect at the entire interface between electrode and switching layer (or the bulk of switching layer). Because the conduction is dependent on the entire area of the device, the ON current scales with the device size [37, 39]. Hence, the area dependency of the operating current is one distinct property of the filament-free type. To further understand these uniform switching phenomena, several fundamental mechanisms based on ionic, electronic/electrostatic, and ferroelectric effect have been suggested. In this Section, we describe diverse types of filament-free switching memristors depending on the main operating principles.

### Ion migration

Migration of ions under the applied electric field is generally considered as one of the main driving forces for the filament-free type of memristors (Fig. 1b). In this type of memristors, a wide range of transition metal-oxide (e.g., TaO_x_, TiO_x_, WO_x_, and HfO_x_) and complex perovskite oxide materials (e.g., Nb-doped SrTiO_3_, LSMO, and PCMO) have been employed as the switching layer[43, 56–63]. Inert (e.g., Pt and Au) or reactive metals (e.g., Ti and Ta) have been utilized for electrode terminals. Basically, interfacial Schottky barrier (*Φ*_B_) between the switching layer and the electrode can form due to the difference in the electron affinity of the switching layer (χ) and the work function of the electrode (*Φ*_M_), simply expressed as *Φ*_B_ = *Φ*_M_ − χ. The *Φ*_B_ can result in a high resistance contact because the major charge carriers are depleted near the interface of the switching layer. This indicates that the height and the width of *Φ*_B_ can determine the overall conduction of the device. Because the *Φ*_B_ can be modulated via the concentration of ions (serve as dopants) [64, 65], the ionic motion controlled by the applied electric field alters the *Φ*_B_, thus causing the switching behavior within the device. For example, in the case of n-type oxide materials, oxygen vacancies (*V*_O_) can be generated by the reactive metal electrode or the non-stochiometric deposition of oxides and they act as the donors. As shown in Fig. 1b, when a positive voltage is applied to the top electrode, the positively charged *V*_O_ can migrate towards the opposite bottom electrode, leading to the accumulation of *V*_O_ near the bottom electrode. This result can lower *Φ*_*B*_ formed at the interface between the switching layer and bottom electrode (e.g., *V*_O_ can act as dopants to lower the *Φ*_*B*_ [65–67]). If it is assumed that the conduction is mainly determined by *Φ*_B_ at the bottom interface, the device switch from its OFF state to its ON state. Conversely, when a negative voltage is applied to the top electrode, the *V*_Os_ move back to the top electrode. Because the *V*_O_ density at the bottom interface is decreased, the lowered *Φ*_B_ recovers the original height, thereby switching the device from its ON state to its OFF state. Likewise, the width of *Φ*_B_ is also dependent on the *V*_O_ concentration given that the depletion region (*W*_*d*_) become narrow or wide according to the equation *W*_d_ ~ *n*^−1/2^ [68]. When the *V*_*Os*_ drift to (repel from) the *Φ*_B_ interface, the *W*_d_ becomes narrower (wider), thus facilitating (inhibiting) the electron tunnelling process through the *Φ*_B_, thereby making the device switching to its ON state (OFF state). In addition, when a sufficient electric field disappears, the accumulated *V*_Os_ at the ON state are prone to spontaneously back diffusion driven by the concentration gradient. This is why the volatility is typically observed in this type of memristors. Consequently, the electric field-driven accumulation and depletion of ions in the vicinity of the interface could drive the homogenous switching events in this type of memristors. Note that the material combinations can affect the role and type of ions and band structure in the devices. For example, in the case of p-type oxide materials, the *V*_Os_ provide the opposite effect on the modulation of *Φ*_B_; namely, the more *V*_Os*`*_ are, the higher the *Φ*_B_ is.

### Intercalation

Reversible intercalation of ions into the lattices provides the basis for the switching mechanism of this type of memristors (Fig. 1c). Generally, ion-blocking metals (e.g., Pt and Au) have been utilized for the two electrodes where the junction is separated by multiphase ion-intercalation materials (e.g., LiCoO_2_ and Li_4_Ti_5_O_12_) [69–73]. In this junction, ions can be inserted into and deserted from the switching layer while maintaining the original crystal structure [74]. If a downward or upward electric field is introduced into the device, cations such as Li^+^ can drift toward the bottom or top electrode while depleted at the opposite electrode. These processes result in the concentration polarization effect observed in various electrochemical systems [75–77]. As the cations are gradually inserted into and deserted from lattices near the electrodes, multiphase polarization can be formed according to the ion concentration [78]. Because the number of free charge carriers in the conduction/valence bands depends on the local ion concentration, switching behavior can be controlled by the degree of concentration polarization if contact resistance dominates the entire resistance of the device [78–80]. For example, as shown in Fig. 1c, when a positive voltage is applied to the top electrode, the cations accumulate near the bottom electrode in which the transition from polarization-poor to polarization-rich phase occurs gradually. Assuming that the bottom interface dominates the overall conduction rather than the top interface, the increase in the cation concentration is able to enhance the charge transport rate at the bottom interface and then the resulting overall resistance can be reduced (i.e., switching from the OFF state to the ON state). Notably, unlike ion migration in the homogeneous switching materials (Sect. 2.1), the multiphase mechanism allows for the inhomogeneity of the ion concentration within the switching layer, even after the removal of the applied programming voltage. Hence, the non-volatile nature can maintain the ON state (intermediate states as well) for a long time of period. By contrast, when a negative voltage is applied into the top electrode, the cations move near the top electrode with the phase redistribution into the original states, thus decreasing the charge transport rate at the bottom interfaces. Subsequently, the device returns into the OFF state. As a result, in this type of memristors, the formation of multiphase based on the electric field-driven ion migration and intercalation can be considered as a primary principle of switching mechanism. Compared to typical ion migration of Sect. 2.1, when the applied voltage disappears, phase distribution altered by inserted ions can maintain almost without the spontaneous relaxation, forming the concentration polarization at each phase, thus leading to nonvolatile multiphase polarization. The nonvolatile multiphase polarization can be explained by the phase-separation system, where the mass transfer is mainly governed by the spatial gradient of the ion’s chemical potential (*μ*), rather than the gradient of ion concentration. Because the phase-separation systems typically exhibit the flat profile of *μ*, a certain multiphase polarization does not return to the original state but maintains almost without the ion movement by the gradient of ion concentration [70, 78]. This behavior cannot be well-fitted by the drift–diffusion model [81]. As a result, the phase separation can enable the coexistence of ion-rich and ion-poor regions, leading to nonvolatile multiphase polarization. Intercalation-based memristors generally suffer from the long switching time at a moderate voltage value. To alleviate this issue, several approaches have been suggested, such as the use of higher ion mobility, the decrease in the thickness of switching layer, and the increase in switching current [70, 82]. For example, ion diffusivity can be improved by using lower-dimensional ion-intercalation materials with reduced diffusion paths (e.g., 1D and 2D materials). This method can mitigate the issue regarding long switching time. Note that it would be not allowable when switching layer is too thin to form the coexistence of different phases.

### Electron trapping/detrapping

Electronic effect based on the trapping and detrapping processes of electrons is another main principle for the filament-free switching memristors (Fig. 1d). There have been various materials and junction structure for the switching layer of this type of memristors, for example, TiO_x_ [83], WO_x_ [84], HfO_x_ [85], Ba_0.7_Sr_0.3_TiO_3_ [86], InGaZnO (IGZO) bilayer [87], Bi_2_S_3_/F-doped SnO_2_ [88], NbO_x_/TiO_x_/NbO_x_ [89], Ta_2_O_5_/Nb_2_O_5−x_/Al_2_O_3−y_[90]. The electric field applied to these junction structures can either trap or detrap electrons when trap sites are distributed within the switching layer (Fig. 1d). Because the number of trapped electrons near/at the interface can alter the *Φ*_B_, this type of memristors switch without the formation of conductive filaments. Figure 1d illustrates a trapping and detrapping mechanism of electrons in case of the oxide material between the two metal electrodes. Herein, we assume that the *Φ*_B_ at the top interface is responsible for the overall conduction. When a positive bias is applied to the top electrode, the trap barriers (*Φ*_trap_) at the top interface can be lowered due to the direction of the electric potential, such that electrons can be readily released from the traps. The detrapping process can reduce the *Φ*_B_ because the total charge in traps near the interface (*Q*) changes the *Φ*_B_ by ~ *qδQ*_*m*_/*ε*, where *q* is elementary charge, *δ* is the distance between the total charge in traps and the interface, *Q*_*m*_ is trapped charge per unit area, and *ε* is the permittivity near the interface [91]. These processes naturally lead to the ON state of the device. However, when a negative bias is applied to the top electrode, the electrons can be injected and captured at the trap sites, thus increasing the number of charged traps. Accordingly, the *Φ*_B_ at the top interface increases and recovers again, which can return the device from the ON state to the OFF state. In addition to this scenario, there is a bulk-limited conduction mechanism in the case of the defect-rich dielectric materials [referred to as space charge limited conduction (SCLC)] [92, 93]. There is an assumption that the interfacial barrier is the Ohmic contact to readily inject electrons into the dielectric layer. At a low bias regime, the current behaviors follow Ohmic conduction because the dielectric relaxation of injected electrons occurs before they reach the anode. Namely, the current is mainly regulated by thermally generated free carriers within the dielectric layer rather than the injected electrons (i. Ohmic region, OFF state) [94]. At a high bias regime, the residence time of injected electrons in the space between two electrodes decreases and equilibrates to space charge distribution. In other words, the transition time of electrons from cathode to anode is too short to be relaxed by the thermally generated carriers, implying the generation of the space charge field. Because the internal electric field induced by the space charge counteracts the electric field applied between electrodes, the current is limited by the space charge [95]. However, because the localized trap sites can capture the electrons and prevent them from traversing the dielectric layer, the total current flow is additionally limited until the traps are all occupied (ii. trap-limited SCLC, OFF state). During this stage, the number of injected electrons rapidly increases and the fermi level (*E*_f_) approaches the edge of conduction band, thus increasing the current exponentially. Eventually, all the trap sites located between two electrodes are filled by injected electrons, which can induce the transition from trap-limited SCLC to trap-free SCLC. Hence, at a higher bias regime, only space charge limited conduction governs the overall current behaviors as the effect of trap sites on the charge transport almost disappears (iii. trap-free SCLC, ON state). When the bias decreases, the electrons can escape from the traps while the free carriers by the thermal excitation become dominant again, returning the device into the OFF state. Notably, because the bulk-limited mechanism originates from the electrical feature of the dielectric layer itself, switching behaviors are observed to be independent on the electrode material and bias polarity [96], indicating that the switching transition between OFF and ON can be generated in the same voltage polarity. Therefore, the conduction behaviors controlled by the charge (de)trapping is responsible for the fundamental switching principle of this type of memristors without the formation of conductive filaments. It should be noted there are several distinguishable differences between the electron (de)trapping near/at the interface and SCLC in the bulk. One is the dependency on the bias polarity. Because *Φ*_B_ formed at interface mainly regulates overall charge transports, *Φ*_B_ modulation based on the electron (de)trapping process results in bipolar switching behavior in memristors, where SET and RESET transition occurs at different voltage polarity regime. However, bias-independent SCLC utilizes Ohmic contacts to facilitate the carrier injection into the bulk, thus inducing unipolar switching event in memristors. Another is the electrode selection. Considering that height (or width) of *Φ*_B_ initially formed at interface can affect the switching behavior, the electrode selection in the interfacial (de)trapping process becomes more crucial than in the SCLC mechanism to control switching characteristics. Further, a threshold switching event is typically observed in the SCLC than in the interfacial trapping and detrapping process. This difference can be simply explained by the transition region from trap-limited to trap-free SCLC. Therefore, the two mechanisms have quite different characteristics. Additionally, similar to the ion migration, this type of memristors is also apt to exhibit a short retention time primarily because of the voltage–time dilemma (e.g., an insufficient barrier height/width and a much higher density of charge carriers compared to the number of traps). In other words, it is difficult to secure long retention time without a large voltage and/or a long switching time, mainly because trapped electrons can jump over and tunnel through the barrier [97]. For example, because a moderate input voltage can trap electrons into relatively low and/or thin trap barrier, a certain electronic state becomes rapidly relax into the original state as the applied voltage disappears. In contrast to filamentary types of memristors, there is almost no thermal feedback process induced by the local Joule heating effect in filament-free types of memristors. This can result in a much less nonlinear kinetics during the switching process, which naturally leads to poor retention time at a short switching time with moderate voltage value.

### Ferroelectric effect

A ferroelectric material may possess multiple spontaneous polarizations without an external electric field, which can be switched by applying a sufficiently high external field [98–100]. Such nonvolatility and tunability of polarization make them attractive for implementing memristive devices, such as ferroelectric tunnel junctions (FTJs) [101–113]. The spontaneous polarization of the ferroelectric material is an electric dipole that builds an electric field in space. Unless both surfaces of the ferroelectric film come into contact with perfect metal electrodes, the depolarization field arises across the film, and the potential varies in the metal over a finite distance from the interface, known as Thomas–Fermi screening length. The potential drop in the top (ψ_TE_) and bottom (ψ_BE_) electrodes, whose signs are always opposite due to opposite charges near the interfaces, effectively changes Φ_B_ at each interface, leading to the average tunneling barrier height change by (ψ_TE_ + ψ_BE_)/2 (Fig. 1e) [114]. For instance, when the screening length of the top electrode is longer than that of the bottom electrode, |ψ_TE_| is larger than |ψ_BE_|. The average barrier height is higher when the head of polarization (positive charge) points toward the bottom electrode, resulting in lower tunneling probability (low conductance state). Therefore, the polarization direction in a ferroelectric layer modulates the conductance of FTJ. The conductance ratio for the ON and OFF states, i.e., tunnel electroresistance (TER), generally increases with the difference in screening lengths between the two electrodes, hence a semiconductor can be employed as an electrode for FTJ to enhance the TER [102]. These polarization-induced tunnel barrier modulation was manifested not only in asymmetric electrode structures but also in composite barrier structures with dielectric layer [109, 115]. The ferroelectric polarization charges at the interface between the dielectric and the ferroelectric layer are partially compensated by the electric displacement from both layers inducing the internal field and the depolarization field, which are oppositely direct to each other. Therefore, the average potential barrier height of the composite barrier is lower (higher) when the polarization heads to the interface (the electrode). A distinctive microstructure, namely, the multi-domain structure, of ferroelectric film enables the stable multi-level conductance of FTJs [103]. Since ferroelectric domains are stable in their respective spontaneous polarization states (e.g., up or down) and can be independently switched in response to an external electric field, the total conductance of FTJ is modulated by the area-portion of up and down domains.

### Phase transition

Phase transition, which involves a change in electrical properties, can significantly alter the conductance of materials (Fig. 1f). Depending on the arrangement of atoms, different phases can coexist in solid form, e.g., anatase and rutile phases in TiO_2_ [116, 117]. Structural changes between phases induce changes in the band structure of the material, resulting in changes in its electrical properties [118–126]. Materials that exhibit a reversible phase transition upon electrical stimulation can be utilized as a memristor. In particular, memristors accompanied by a phase transition involving ion migration are categorized as phase transition memristors in this Review. It should be noted here that if a material exhibits phase transition and if the change in electronic band structure is dominated by Mott physics, it will be classified as a Mott memristor and introduced in the next section. As mentioned above, the *Φ*_*B*_ is formed at the interfaces between the switching layer and the electrodes. In general, the bandgap of an insulator is larger than that of a semiconductor, so the *Φ*_*B*_ is also higher when the switching layer is an insulator, resulting in a lower conductivity. Even more, when an insulating or semiconducting material changes to a metallic phase, it can remove most of the *Φ*_*B*_ and increase the conductance of the memristor. While the proportion of regions with different bulk conductivities in a matrix can affect the conductance of a device, in memristors with thin switching layers, carrier injection into the interface has a more significant impact than bulk conductivity. Introducing defects to the matrix by intercalation, chemical doping, or removal of atoms can readily modulate the energy landscape of the system, leading to phase transitions. Mobile ions driven by an external electric field are attracted to one interface of the two-terminal memristor, inducing a phase transition in the matrix on that side of the interface. For instance, transition metal dichalcogenides, such as MoS_2_ and WSe_2_, favor the metallic 1 T’ phase over the insulating 2H phase when lithium ions are incorporated into the matrix [118, 119, 121]. In addition, anion stoichiometry modulation in transition metal oxide perovskite, such as SrFeO_3−x_ and SrCoO_3−x_, induces phase transition from metallic oxygen-deficient conducting perovskite structure (ABO_3−x_) to insulating brownmillerite structure (ABO_2.5_) [122–126].

### Mott transition

The Mott material, which possesses split energy bands in the transition metal d- or f-orbitals due to strong electron–electron interaction, manifests electrical properties different from those expected by classical band theory (Fig. 1g) [127–129]. Due to the energy difference between the split energy bands, called the Mott–Hubbard gap, a material that is expected to be metallic according to classical band theory due to a partially filled d- or f-orbital exhibits insulating properties since its d- or f-bands split into a fully occupied lower Hubbard band and an empty upper Hubbard band, similar to band insulator in classical band theory. The Mott insulator can be transformed into metal, i.e., insulator to metal transition (IMT), through temperature [130, 131], pressure [132–134], voltage [135–137], or charge doping [138, 139], accompanying electronic structure change, such as the width of energy band change or Fermi level shift. However, methods to control temperature and pressure in integrated electronic devices are elusive. On the other hand, charge doping-induced IMT, so-called filling-controlled Mott IMT, can result from electron doping into the fully empty band or hole doping into the fully occupied band. For instance, the perovskite structure materials with trivalent transition metal on B sites (ABO_3_, B = Ti, Ni, Mn) can manifest IMT by employing oxygen stoichiometry change. Excess oxygen in the lattice further oxidizes the B site ion, which reduces the electrons in the transition metal d- or f-orbitals, and thus, holes are doped into the lower Hubbard band, making the Mott insulator into a p-type conductor. Similarly, hydrogen ion or *V*_*O*_ doping can change the valence states of transition metal ions, filling the upper Hubbard band; thus, the Mott insulator becomes an n-type conductor. The increase or decrease of the valence state induces the change of electron interaction in the orbitals, resulting in the energy gap modulation as well as electron or hole doping. As discussed in Sect. 2.1, ions are migrated by an external electric field and accumulated near the electrode, enabling filling-controlled Mott IMT to incur at the region in a high concentration of ions. Therefore, the filling-controlled Mott transition is a feasible operating mechanism for the non-filament switching memristors. The different roles of ions, especially *V*_*O*_ in oxides, in the typical band insulators and the Mott insulators should be pointed out. The oxygen deficiency in the Mott insulators dominantly affects the valence states of transition metal, the filling state of d- or f-orbitals, whereas *V*_*O*_ in the band insulator forms the energy level between the band gap, altering *E*_*F*_ of the switching layer.

## Recent studies on filament-free switching memristors

In this Section, we introduce and review the representative efforts on filament-free switching memristors distinguished by their switching principles. Previously, a few prior reviews have addressed non-filamentary switching memristors with an emphasis on switching materials [37, 39]. Here, we mainly focus on the various junction structures, their switching mechanism, and their switching behaviors that can be exploited as computational primitives. This is because implementation of memristive computing systems inevitably require understanding, design rule, and engineering methods regarding different material combinations and device structures according to the main application (e.g., bio-realistic neuron/synapse, time processing, and physical matrix calculation). The representative examples in Sect. 3 are worthwhile because they provide different junction structures and utilize them for different computing applications. Note that it does not mean that other references not selected in Sect. 3 are less important. Note that Table 2 presents a summary of various filament-free switching memristors and their structural and electrical characteristics, which could facilitate a comprehensive understanding of their essential switching performances.

**Table 2:** A summary table of various filament-free switching memristors along with key parameters

Type	Junction structure	Device size (μm^2^)	Operating voltage (V)	Endurance (cycles)	Retention (s)	ON/OFF ratio	Uniformity (d2d/c2c)	Refs.
Ion migration	Pt/anodized TiO_x_/Ti	5 × 5–50 × 50	− 2.5 to 3	5 × 10^6^ pulses	4 × 10^–2^	< 10	3.87%/1.39%	[57]
	Pt/a-GaO_x_/ITO	~ 1–30,000	− 2 to 2	–	–	< 10	–/Good	[56]
	TiO_x_/TiO_2−x_/YSZ/TiO_2_/Pt	–	− 1.5 to 1.5	3 × 10^8^ pulses	10^7^	< 3	–/Good	[58]
	Au/MAPbI_3_/ITO	500 × 500	− 1 to 1	–	< 10^–2^	< 10	–/Good	[59]
	Pt/TiO_2_/TiO_2−x_/Pt	0.05 × 0.05	− 2 to 2	–	NA	~ 10^3^	–/Good	[60]
	Au/Nb:SrTiO_3_	π × 150 × 150	− 6 to 3	10^4^	10^4^	> 10^4^	Good/Good	[61]
	Pt/TaO_y_/nanoporous TaO_x_/Ta	π × 100 × 100	− 4 to 4	5 × 10^3^	1.2 × 10^4^	< 10^2^	< 59%/Good	[63]
Intercalation	Au/Li_x_CoO_2_/SiO_x_/TiO_2_/p^++^-Si	300 × 300	− 5 to 5	> 2 × 10^3^	–	< 10	–/Good	[69]
	Au/Li_x_CoO_2_/doped-Si	10 × 10–500 × 500	− 7 to 7	> 2 × 10^2^	> 3 × 10^2^	> 10^3^	–	[71]
	Cr/LiCoO2/LiPON/a-Si/TiN	300 × 300	− 10 to 10	3 × 10^3^ pulses	> 10^3^	–	–	[72]
	Planar device Li_1−x_NbO_2_ with Ti and Al contacts	–	− 10 to 10	–	> 2 × 10^3^	> 20	–/Good	[73]
Electron trapping /detrapping	Au/TiO_2_ nanorod array/FTO	π × 750 × 750	− 4 to 2	–	< 2	> 10^2^	–	[83]
	Au/WO_3_/FTO	50 × 50	− 3 to 3	10^3^ pulses	10^4^	< 10	–/Good	[84]
	Ti/HfO2/Pt	2 × 2–20 × 20	− 3 to 2	10^3^	10^5^	< 10^2^	Good/Good	[85]
	Planar device Ba_0.7_Sr_0.3_TiO_3_ with Pt contacts	–	− 90 to 90	–	2.5 × 10^2^	< 10	–	[86]
	Au/O_D_-IGZO/O_R_-IGZO/Pt	π × 50 × 50	− 2 to 2	2 × 10^3^ pulses	10^4^	< 3	–/Good	[87]
	Ag/Bi_2_S_3_ nano-network/FTO	π × 400 × 400	− 1 to 1	–	–	< 10	–	[88]
	Pt/NbO_x_/TiO_y_/NbO_x_/TiN	π × 0.015 × 0.015	− 10 to 10	5 × 10^3^	3 × 10^3^	> 10^2^	–/Good	[89]
	Pt/Ta_2_O_5_/Nb_2_O_5−x_/Al_2_O_3-y_/Ti	5 × 5	− 10 to 10	10^5^	2 × 10^5^	> 8 × 10^3^	Good/Good	[90]
Ferroelectric polarization	W/Zr:HfO_2_/SiO_2_/doped Si	π × 20 × 20–π × 70 × 70	− 3 to 3	10^3^	10^4^	> 2 × 10^2^	Good/Good	[102]
	Co/BaTiO_3_/La_0.67_Sr_0.33_MnO_3_/NdGaO_3_	π × 0.175 × 0.175	− 5.6 to 4.2	–	–	3 × 10^2^	–	[103]
	TiN/HfSiO_2_/SiO_2_/bottom electrode	0.15 × 0.15–0.5 × 0.5	− 4.4 to 4.4	–	–	> 3	Good/Good	[104]
	Cr/CuInP_2_S_6_/Graphene	–	− 5.5 to 4.5	5 × 10^3^	> 2 × 10^4^	10^6^	–/Good	[105]
	Ag/BaTiO_3_/Nb:SrTiO_3_	π × 20 × 20	− 3.4 to 3	10^8^	10^4^	> 10^2^	–/Good	[106]
	Ag/PbZr_0.52_Ti_0.48_O_3_/ Nb:SrTiO_3_	π × 50 × 50	− 3.8 to 4	10^9^	10^4^	> 10^2^	–/2.06%	[107]
	Mo/(Hf,Zr)O_2_/TiON/TiN	20 × 20–100 × 100	− 2 to 2	10^8^	3 × 10^4^	< 10	3.2%/3.6%	[108]
	Ti/BaTiO_3_/SrTiO_3_/SrRuO_3_	π × 0.25 × 0.25	− 8 to 8	2 × 10^3^	–	10^4^	–	[109]
Phase transition	Planar device Mos2 with Ti contacts	–	− 50 to 50	5 × 10^3^ pulses	–	< 3	–	[118]
	Planar device Li_x_Mos_2_ with Au contacts	–	− 6 to 6	4 × 10^4^ pulses	> 7 × 10^3^	< 10	–	[119]
	Ti/Al_2_O_3_/MoTe_2_/Au	0.36 × 0.39	− 3 to 3.5	–	10^3^	> 10^5^	–/Good	[120]
	Ag/MoS_2_/Ag	–	− 0.2 to 0.2	10^3^	–	10^3^	–/Good	[122]
	Au/SrCoO_x_/Nb:SrTiO_3_	π × 100 × 100	− 5.5 to 1	7 × 10^2^	10^4^	> 10^3^	Good/Good	[123]
	Au/SrCoO_x_/SrRuO_3_	4 × 4–100 × 100	− 3 to 3	10^8^	3 × 10^3^	> 10	Good/Good	[124]
	Au/SrFeO_x_/SrRuO_3_	π × 50 × 50	− 4 to 4	6 × 10^6^	< 2	> 10	35%/22%	[125]
	PT/α-MoO_3_/SrCoO_2.5_/Nb:SrTiO_3_	π × 25 × 25	− 3 to 3	5 × 10^4^	> 10^3^	< 10	Good/Good	[126]
Filling-controlled Mott transition	Planar device NdNiO_3_ with Pt contacts	–	− 8 to 8	3.6 × 10^3^ pulses	> 10^3^	< 10	Good/Good	[138]
	Planar device NdNiO_3_ or SmNiO_3_ with Au and Pd contacts	–	− 8.1 to 8.1	10^6^	4 × 10^4^	10	–/Good	[139]
	Pt/GdNiO_3−x_/Nb:SrTiO_3_	70 × 70	− 5 to 5	10^4^	> 3 × 10^4^	> 10^3^	Good/Good	[141]
	W probe/La_2_Ti_2_O_7−x_/TiN	–	− 2.5 to 2.5	10^3^	–	< 10	–/Good	[142]

Note that c2c and d2d in the column of uniformity indicate cycle-to-cycle and device-to-device variation, where the coefficient of deviation (standard deviation/mean × 100%) is used when available. Otherwise, the uniformity is represented as good and poor based on how identical the switching behaviors are at switching cycle (c2c) and each device (d2d)

### Ion migration-based memristors

**Fig. 2:**
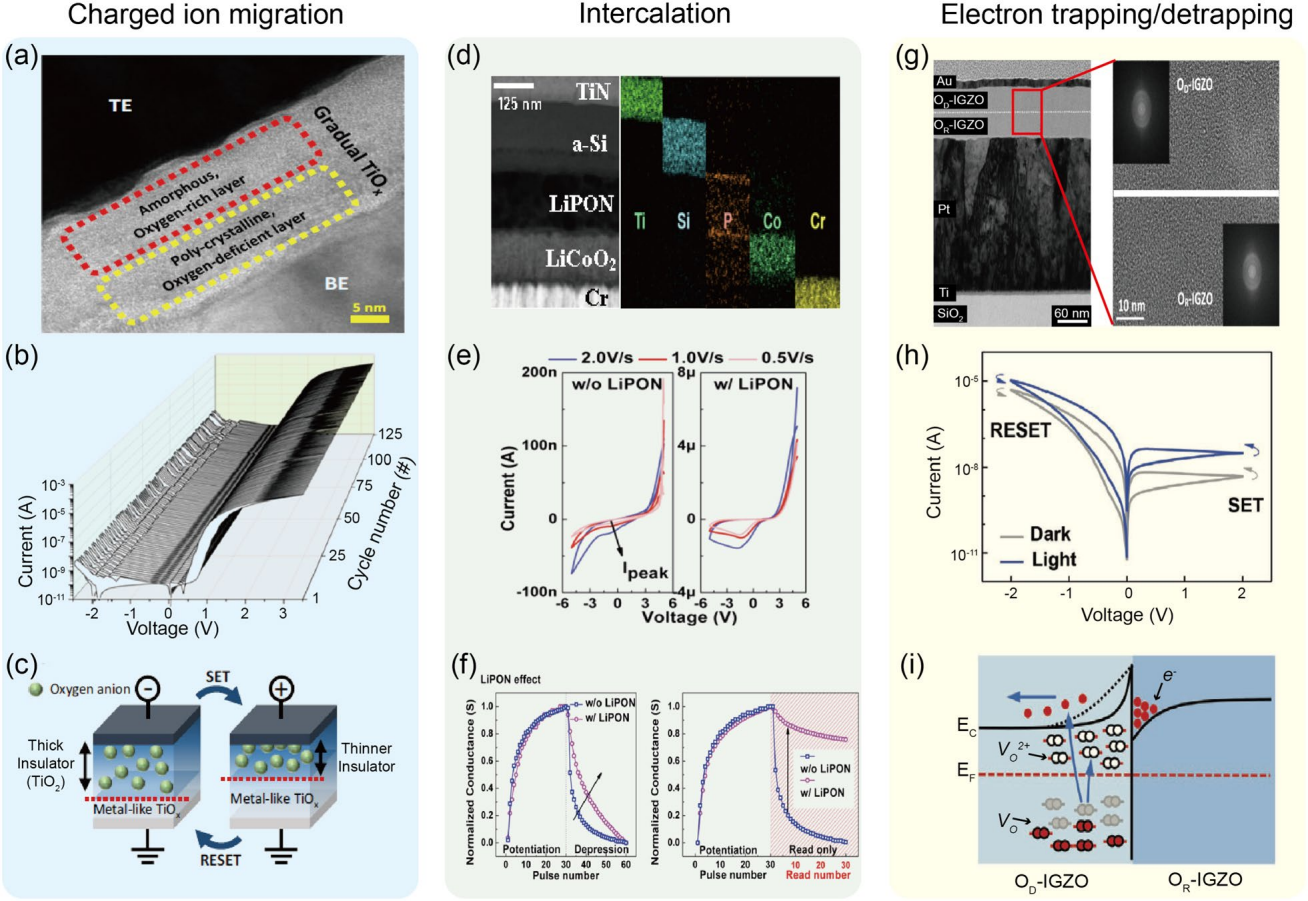
Experimental demonstrations of filament-free switching memristors. **a**–**c** A ion migration-based nonfilamentary memristor. A cross-sectional TEM image of the device structure (**a**), consecutive *I*-*V* switching curves (**b**), and schematic illustrations of the suggested switching mechanism (**c**). Reproduced with permission from Ref. [57]. Copyright 2022, Springer Nature. **d**–**f** A intercalation-based nonfilamentary memristor. Cross-sectional TEM and EDX images of the device structure (**d**), *I-V* switching curves with(out) LiPON (**e**), and plots of normalized conductance with(out) LiPON as functions of potentiating/depressing pulses (left) and potentiating/reading pulses (right) (**f**). Reproduced with permission from Ref. [72]. Copyright 2019, IEEE. **g**–**i** An electron trapping/detrapping-based nonfilamentary memristor. A cross-sectional TEM image of the device structure with the enlarged image (**g**), *I-V* switching curves under dark and light environments (**h**), and a band diagram (**i**). Reproduced with permission from Ref. [87]. Copyright 2020, John Wiley and Sons

The modulation of interfacial barriers based on ion migration is responsible for this type of memristors, with their main switching principle explained in detail in Sect. 2.1. Recently, Park et al suggested a Pt/TiO_*x*_/Ti junction structure as a non-filamentary memristor based on ion migration [57], as shown in Fig. 2a–c. In this junction, they utilized an anodization process to form the TiO_*x*_ layer from the Ti bottom electrode. Because the anodization oxidizes metal from the top side to the bottom side (i.e., top-down oxidation), the anodized oxide layer has a gradual oxidation state. Figure 2a shows a cross-sectional transmission electron microscopy (TEM) image of the Pt/TiO_*x*_/Ti memristor, where the TiO_*x*_ is observed to be gradually oxidized along the depth direction. The anodized TiO_*x*_ layer was also investigated by utilizing the time-of-flight secondary ion mass spectrometer (TOF-SIMS) method, confirming that a gradual increase (decrease) in the oxygen (Ti) concentration from the bottom to the top interface. Figure 2b shows current-voltage (*I*-*V*) curves of the memristor during 125 cycles without the need of the initial electroforming process. Figure 2c illustrates schematic diagrams of suggested operating principles in the device. Negatively charged oxygen anions induced by the anodization could migrate into the top electrode via the application of positive voltage into the top Pt electrode, which reduced the effective thickness of the insulating oxygen-rich region within the switching layer. Hence, the overall conductance increased, resulting in the ON state of the device at the positive voltage regime (Fig. 2b and c). Conversely, the oxygen anions moved back into the bottom electrodes through the application of negative voltage into the top electrode, while the effective thickness of the insulating oxide layer was increased. The conductance state returned to the OFF state during the negative voltage regime (Fig. 2b and c). Further, this device was observed to spontaneously return to the OFF state unless the external electrical inputs were maintained to the device. This self-decaying nature might be attributed to the small activation energy of oxygen diffusion of the gradual TiO_*x*_ (0.21 eV) than that of rutile TiO_2_ (1.05 eV). In other words, the oxygen ions may readily overcome the local barriers even under zero electric field, leading to the short-term memory effect. Based on the volatile property, the device wired with a series resistor and a parallel capacitor was proposed as a leaky integrate-and-fire (LIF) artificial neuron. Note that further descriptions and discussions on artificial neuron applications will be presented in Sect. 4. In fact, a similar anodization process had been employed for a Pt/TaO_*y*_/nanoporous TaO_*x*_/Ta memristor in which a fluorine-embedded acid solution was utilized to form the nanopores inside the TaO_*x*_ layer as well as the depth-dependent gradual oxidation during the anodization process. The nanoporous TaO_*x*_ memristor exhibited a lower switching current and a better retention performance, which will be further discussed in Sect. 4.5. This difference might be explained by the existence of nanopores with its high oxidation and charge/ion trapping ability. Because the device showed a long-term memory, synaptic functionalities such as long-term plasticity and spiking-timing-dependent plasticity had been experimentally demonstrated with it (details in Sect. 4.1).

Moreover, Yang et al. reported an ion migration-based memristor with a stacked ITO/CH_3_NH_3_PbI_3_/Au junction structure [59]. The device showed a threshold-like switching behaviors regardless of the voltage polarity. For example, when a small voltage was applied to the Au bottom electrode, it was unlikely that charge carriers could overcome the interfacial barrier due to the relatively weak electric field. As the applied voltage increased, positive ions (e.g., MA^+^) could drift into the ITO/MAPbI_3_ top interface whereas negative ions (e.g., I^−^) into the MAPbI_3_/Au bottom interface. Then, a self-doping effect was generated near both interfaces, which has been also investigated in other previous works. This self-doping process enabled the modulation of interfacial barrier due to the change in *E*_*F*_ of the switching layer, which could facilitate the charge transport and increase the overall current level (i.e., ON state). When the input voltage became either small or absent, the current was reduced because concentration gradient of the ions and the corresponding built-in potential move the ions away from the interfaces and then recovered the interfacial barriers. Considering that the minimum current was observed at non-zero voltage region, the built-in potential might offset the external electric field. Further, the authors performed an in-situ Kelvin probe force microscope (KPFM) by using the lateral junction structure in order to identify the self-doping effect inside the switching layer according to the direction of the applied electric field. They verified that the *E*_*F*_ shifted toward the conduction and valence bands by the n- and p-doping effects, respectively. However, there is a possibility that the self-doping effect might originate from other fundamentals such as electron trapping/detrapping process. Consequently, a simple LIF artificial neuron combined with external peripheral circuits has been demonstrated by utilizing the volatile and threshold switching behaviors.

Further, Aoki et al. fabricated a Pt/GaO_*x*_/ITO junction structure for a filament-free switching memristor [56]. In this junction, current was observed to decrease (increase) when a positive (negative) bias was applied to the top Pt electrode. The change in the current value might be ascribed to the homogeneous enrichment and depletion of the oxygen ions near the top electrode in response to the bias polarity, which was experimentally demonstrated by TEM and energy dispersive X-ray spectroscopy (EDX) results. It was speculated that the switching region lied in the bulk GaO_*x*_ rather than the Pt/GaO_*x*_ interface. To support this, a numerical simulation for the homogenous switching have been presented based on the modulation of the carrier concentration profiles in the amorphous n-type semiconductor by redistribution of the *V*_*O*_. This simulation result verified that the electronic conductivity was associated with the distribution of *V*_*O*_ concentration (i.e., donor concentration) in the GaO_*x*_. This result could be explained by the concept of mobility edge that the *E*_*F*_ move into either the localized or delocalized states according to the bias-dependent carrier density [140]. Further, considering that the oxygen concentration profile was actively modulated via the bias polarity within the depth range of 10 nm underneath the top electrode, the ion drift and diffusion motion as well as the oxygen concentration profile could act as the internal conductance states of GaO_*x*_ layer. These principles were related to the switching event in the device. Hence, this type of memristor could be a device platform for future neuromorphic applications because of its dynamical behavior and multilevel states of conductance.

### Intercalation-based memristors

Intercalation and migration of ions are recognized as a major switching principle for this type of memristors, and detailed operating processes have already been described in Sect. 2.2. Choi et al. recently proposed a TiN/a-Si/LiPON/LiCoO_2_/Cr as a non-filamentary switching memristor [72]. Figure 2d shows the cross-sectional TEM and EDX images of the fabricated device. The LiPON layer was employed as a solid-state electrolyte through which ions can migrate between a-Si and LiCoO_2_. The TiN and Cr electrodes were utilized to prevent reactions with other layers. When a positive voltage applied to the bottom electrode, the ionized Li moved toward the top electrode under the electric field and then the cations were stored in the a-Si anode layer via electrochemical reaction. As a result, the conductance of the device increased because intercalated Li ions altered the electronic distribution. When a negative voltage was applied to the bottom electrode, the cations moved back into the LiCoO_2_ layer from the a-Si layer. To further optimize the switching characteristics, the LiPON interlayer between LiCoO_2_ and a-Si was adopted to control the ion motion. As shown in Fig. 2e, when a LiPON interlayer was inserted, the current was observed to increase despite the application of the same operating voltage. This verified that the LiPON assisted the migration of the cations. Moreover, as shown in Fig. 2f, an abrupt decrease in the conductance and the poor retention property were alleviated to some degree when using the LiPON layer. Because the interlayer could alleviate self-injection phenomenon from the a-Si layer to the LiCoO_2_ layer, the stability of migrated cations might be maintained even after the removal of the external bias. These results indicated that the porosity and the interlayer insertion were able to control the migration rate of Li ions. Based on the optimal layers and the junction design, the device has been suggested as an artificial synapse with acceptable endurance and retention characteristics.

Mai et al. reported a homogenous switching memristor with Au/Li_*x*_CoO_*2*_/doped-Si junction structure [71]. The fabricated device exhibited a high ON-OFF ratio (> 10^4^) and multiple conductance states without the completion of initial electroforming process. To confirm the homogenous switching in the device, the number of voltage pulses required for the ON-transition process was investigated as a function of electrode size. As the size decreased, the number of voltage pulses was observed to dramatically decrease, indicating the typical area-dependency, namely, an experimental support for filament-free switching. In this junction, Li ions could move downward, formed Li_*x*_Si complexes in the doped-Si, and then generated electromotive force (EMF), when a negative voltage was biased into the bottom electrode. To facilitate the migration of Li ions, solid-state electrolyte SiO_*x*_ layer at the Li_*x*_CoO_2_/Si interface (> 9 nm) was formed by the thermal oxidation process. Interestingly, it is known that the x in the Li_*x*_CoO_2_ can determine the electrical conductivity. For example, the Li_*x*_CoO_2_ can act as an insulator and a conductor when *x* > 0.94 and *x* < 0.75, respectively. Also, the coexistence of insulating and conducting phases is possible when 0.75 < *x* < 0.94. From the X-ray diffraction results, the *x* was found to be ~ 0.95 in the pristine Li_*x*_CoO_2_. When a negative voltage was biased into the bottom electrode, Co could be oxidized whereas Li ions move into the Si electrode with the formation of Li_*x*_Si complexes in Si. Subsequently, the *x* value of the Li_*x*_CoO_2_ could be reduced by making the device ON state. After the applied voltage was removed, the EMF value was observed to spontaneously discharge while maintaining the ON state. This result might be attributed to the limited re-insertion of Li ions into Li_*x*_CoO_2_ and the Li ions trapped in the SiO_2_ layer. Hence, a positive voltage was biased into the bottom electrode to perform the OFF-transition process. Consequently, the researchers have suggested the availability of the device to cognitive computation by mimicking different synapse-like behaviors.

Tian et al. fabricated a filament-free switching memristor with a Pt/Li_4 + 3*x*_Ti_5_O_12_ (LTO)/Pt junction structure (*x* was either ~ 0 or ~ 1) [70]. In this junction, the migration of Li ions driven by electric field could be limited at either Pt/LTO or LTO/Pt interface due to the ion blocking Pt electrode. Because the effective conductivity of LTOs was confirmed to be lower than the bulk of them in the literature, it was likely that transport rate of electrons at each interface governs the overall conduction. In this sense, the interfacial transport rate could be influenced by the degree of phase redistribution and different combination of electrodes. For example, when a positive voltage was applied into the top Pt electrode, Li ions can move into the bottom electrode, altering the phase distribution induced by the concentration polarization, thus affecting the interfacial electron transfer rate. In addition to this, if the bottom interface is more dominant, the electrical conductivity of the device could be increased. Then, a phase-field model that anticipates the formation of conductive filament after the initial electroforming process and its based volatile (or irreversible) switching characteristics due to the quick dissolution of filaments when the applied voltage disappears (or insolubleness when a reverse voltage appears) has been amended by reflecting the transport principle of ion and electron and their non-equilibrium thermodynamics. Based on the redesigned model, several guidelines to further develop the intercalation-based memristors have been predicted. For example, the fabricated LTO memristor exhibited a too long switching time (the order of seconds). Regarding this issue, it was expected that other intercalation materials with a higher ion mobility, a decrease in the thickness, and an increase in the operating current could reduce the switching speed due to the enhanced ion diffusivity. With these results, several approaches to device design and optimization have been suggested for future memory and its based computing application.

### Electron trapping/detrapping-based memristors

Purely electronic phenomena provide an underlying mechanism for this type of filament-free switching memristors, as detailed in Sect. 2.3. Hu et al. reported an all-optically controlled memristor by employing a bi-layered oxide sandwiched between top Au and bottom Pt electrode, where the bilayer structure consisted of an oxygen-deficient IGZO (O_D_-IGZO) and oxygen-rich IGZO (O_R_-IGZO) as shown in Fig. 2g [87]. The fabricated device showed a typical bipolar switching behavior in dark environment (Fig. 2h). Namely, a positive voltage enabled the conductance transition from the OFF state to the ON state, whereas a negative voltage induced the reverse transition. This could be attributed to the formation of a potential barrier on the O_D_-IGZO side and a potential well on the O_D_-IGZO side since a built-in electric field was formed due to the electron diffusion from O_D_-IGZO to O_R_-IGZO (Fig. 2i). Note that the O_D_-IGZO and O_R_-IGZO was deposited via sputter with Ar and Ar + O_2_ gas environment. During the application of positive voltage onto the top Au electrode, electrons could be released from neutral *V*_Os_ near the interfacial barrier region, thereby leaving ionized *V*_*Os*_* behind*. Thus, the width of interfacial barrier was reduced, leading to an increase in tunneling current over the junction structure. In contrast, during the application of negative voltage onto the top Au electrode, electrons could be captured at the ionized *V*_*Os*_, which widened the width of the interfacial barrier while limiting the electron tunneling process through the barrier. As shown in Fig. 2h, the current level was observed to increase in light environment. Because *V*_*Os*_ located around the O_D_-IGZE/O_R_-IGZO interface were ionized due to the light illumination, the width of interfacial barrier decreased, which facilitated the electron transport within the device. This ionization of *V*_Os_ also resulted in multiple conductance level in response to the light of different wavelengths (420–1000 nm). Notably, in this junction structure, a decrease in the conductance value was obtained by using near-infrared light when the initial ON-transition occurred through a relatively short-wavelength light (e.g., blue). Because a light input with a shorter wavelength was apt to ionize more *Vos* at the O_D_-IGZO/O_R_-IGZO interface, electrons in the potential well located at the O_R_-IGZO side could transport into the conduction band of the O_D_-IGZO via tunneling through or jumping over the barrier, rather than the ionization of *V*_*Os*_. Then, it was likely that the electron trapping process neutralized the charged *V*_*Os*_ while increasing the width of the interfacial barrier. Based on the characteristics, the authors demonstrated an all-optically controlled memristor by using blue and near-infrared light pulses, suggesting it as an artificial synapse.

Moreover, Kim et al. demonstrated a self-rectifying analog charge trap memristor based on a Pt/Ta_2_O_5_/Nb_2_O_5−x_/Al_2_O_3−y_/Ti [90]. Multiple conductance states were obtained and attributed to the charge trapping into the defect states of the Nb_2_O_5−x_ layer. For example, in the initial OFF state, *Φ*_*B*_ at the Ta_2_O_5_/Nb_2_O_5−x_ interface was high because the deep traps were empty. When a sufficient positive bias is applied into the top Pt electrode, the electrons were captured at the deep traps, which could induce the internal electric field and bend the conduction band. As a result, the lowered *Φ*_*B*_ at the Ta_2_O_5_/Nb_2_O_5−x_ interface was able to increase the overall conduction within the device. Simultaneously, the ON-transition process could be stopped at a certain positive programming voltage, leading to multiple conductance states because the shallow trap levels moved toward the *E*_*F*_ of the Ti electrode and then it limited additional electron trapping process. Also, the high band offset at the Ta_2_O_5_/Nb_2_O_5−x_ and Nb_2_O_5−x_/Al_2_O_3−y_ interfaces, which stabilized the trapped electrons and also suppressed spontaneous trapping processes, enabled the acceptable retention capability. Conversely, a sufficient negative bias could release the electrons at the trap sites and decrease the conductance state through the reverse sequences. Regarding the self-rectifying property, the authors supposed that Al_2_O_3−y_ layer may suppress the current response at the negative bias region after performing comparison between different junction structures such as Pt/Ta_2_O_5_/Nb_2_O_5−x_/Ti, Pt/Nb_2_O_5−x_/Al_2_O_3−y_/Ti, and Pt/Ta_2_O_5_/Nb_2_O_5−x_/Al_2_O_3−y_/Ti. Although the retention property was improved due to the barrier layers at the both electrodes, the operating voltage was relatively high (> 6 V) that might be associated with the voltage-time dilemma. Then, they expanded the fabricated device into a 32 × 32 crossbar array to identify the energy-efficient computing applications with a suggested parallel programming scheme.

Further, Saitoh et al. demonstrated a Pt/CoO/ITO memristor based on the SCLC mechanism [93]. The device showed a threshold switching behavior between OFF and ON state in the same bias polarity. As described in Sect. 2.3, the linear conduction (*I* $\propto$ *V*) was observed at low bias regime (0–0.6 V) because the effect of injected charge carriers on the electrical conductivity was negligible. As the amplitude of bias increased (1.1–1.68 V), the transition time of injected charged carriers became too short to be relaxed by the thermally generated carriers, which could increase the current level although limited by the existence of trap sites within the CoO layer (i.e., trap-limited SCLC). Note that if the density of the injected carriers is lower than that of the thermally generated carriers, the injected carriers could be redistributed into the thermally generated carriers due to the conservation of electric charge neutrality. When the bias was further increased to ~ 2 V, the current level was observed to jump up by the order of two, indicating the transition between trap-limited SCLC and trap-free SCLC. After then, the current follows ~ *V*^2^ (i.e., the trap-free SCLC). As the amplitude of bias decreased to ~ 0.92 V, the trap sites would become empty again as the injected charged carriers escape from the traps, thus returning the device into OFF state. By utilizing the threshold switching behaviors, the researcher simply demonstrated an one-selector-one-memristor configuration, where the device was utilized as a selector. The details of the selector application will be discussed later (Sect. 4.4).

### Ferroelectric effect-based memristors

The average tunneling barrier modulation due to the polarization switching in the ferroelectric layer is a major operating mechanism for this type of memristors, and specific principles have been discussed in Sect. 2.4. Chanthbouala et al. reported the analog ferroelectric memristor based on an Au/Co/BaTiO_3_ 2 nm/La_0.67_Sr_0.33_MnO_3_ 30 nm/NdGaO_3_ structure [103]. Their research is worth revisiting, although it was published almost a decade ago. With a pulse width of 2 ns and voltage swept between − 5.6 V and + 4.2 V, hysteretic resistance versus voltage curves between low (R_ON_ = 1.6×10^5^ Ω) and high (R_OFF_ = 4.6 × 10^7^ Ω) resistance states are observed in the 350 nm diameter device with a TER of about 300. Piezoresponse force microscopy on devices with various resistance states confirmed the correlation between the resistance and microscopic domain configuration. Initially, the device was programmed into the lowest resistance state by pre-poling with a negative bias ensuring virtually homogeneous up-polarized states. Applying consecutive positive incremental step pulses on the device, nucleation and expansion of reverse domains were observed, increasing the fraction of the down-polarized region. As a result of the PFM analysis, the resistance of the device exhibits an evident trend associated with the relative fraction of the downward domain. A parallel resistance model described the relationship between the resistance and the fraction, considering the parallel current path with different resistance in the upward and downward regions (R^−11^ = s × R_OFF_^−1^ + (1−s) × R_ON_^−1^, where s is the fraction of the downward domain). Thus the memeristive behavior of a ferroelectric memristor is attributed to the domain structure following nucleation and growth physics, suggesting the stable multi-level characteristics from the multi-domain nature of the ferroelectrics.

**Fig. 3:**
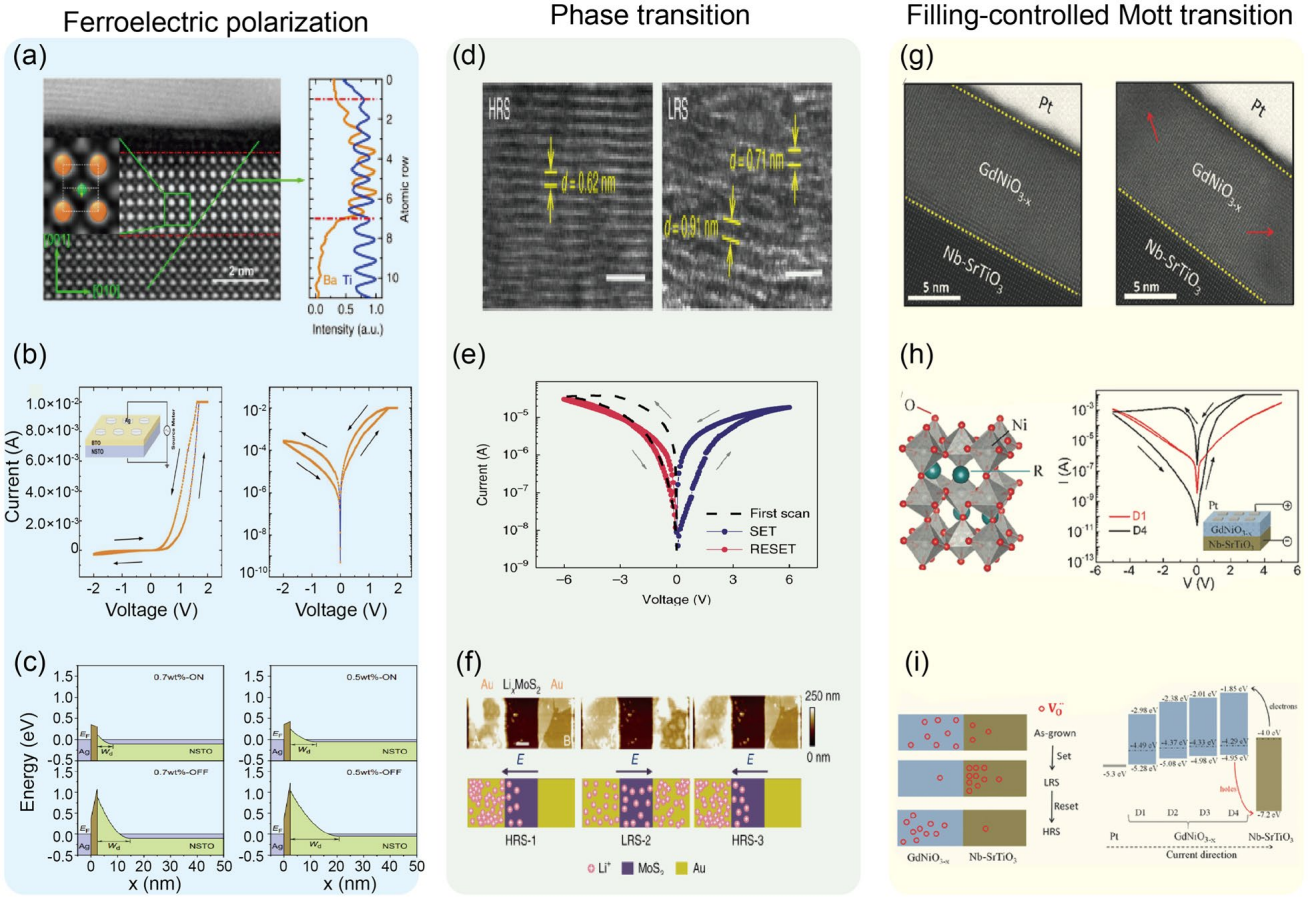
Experimental demonstrations of filament-free switching memristors. **a**–**c** A nonfilamentary memristor based on the ferroelectric effect. Clear lattice fringe from the device structure (**a**), *I-V* hysteresis (**b**), and the modulation of the barrier structure according to the polarization direction (**c**). Reproduced with permission from Ref. [106]. Copyright 2020, Springer Nature. **d**–f A phase transition-based nonfilamentary memristor. Cross-sectional TEM images of MoS_2_ 2H phase and lithiated MoS2 1 T phase (**d**), *I-V* switching curves accompanying first negative sweep (**e**), and AFM topography and schematics of Li^+^ ion distribution for device states (**f**). Reproduced with permission from Ref. [119]. Copyright 2019, Springer Nature. **g**–**i** A Filling-controlled Mott transition-based nonfilamentary memristor. Cross-sectional TEM images of the device structure **g**, *I-V* switching curves for different device fabrication conditions (**h**), and the switching mechanism based on bandgap and Fermi level change of GdNiO_3−x_ layer (**i**). Reproduced with permission from Ref. [141]. Copyright 2017, John Wiley and Sons

In addition, Ma et al. demonstrated the sub-nanosecond operation of the ferroelectric memristor [106]. The Ag/BaTiO_3_ 2.4 nm/Nb:SrTiO_3_ structure is shown in Fig. 3a, and enlarged TEM image inset figure showed that the Ti ion (green circle) is slightly mispositioned from the center of the lattice (center of orange circles), suggesting spontaneous polarization of the ferroelectric BaTiO3 film. The hysteresis direction in the current versus voltage curve in Fig. 3b corresponds to the polarization switching of the BaTiO_3_ layer on the n-type Nb:SrTiO_3_ bottom electrode. The average barrier height and width decrease when the ferroelectric polarization points to the substrate (Fig. 3c), increasing the conductance of the junction. The rate of resistance change was found to follow Merz’s law, which describes the relationship between ferroelectric switching speed and electric field, indicating that the memristive behavior originates from ferroelectric switching.

While high-quality epitaxial thin-film growth techniques have been utilized to fabricate FTJs with conventional perovskite-structured ferroelectric materials, HfO_2_-based ferroelectric thin films have been realized as FTJs through atomic layer deposition (ALD), which is favorable for mass production. Another advantage of ALD is conformal deposition on the surface of a three-dimensional structure, resulting in the fabrication of three-dimensional devices with ALD film and an array of them. Chen et al. reported TiN/Hf_0.5_Zr_0.5_O_2_ 9 nm/Pt FTJ in a three-dimensional structure [110]. Thanks to matured ALD process of HfO_2_, the FTJs were successfully fabricated on the sidewall of the vertical pillar, suggesting an area-effective three-dimensional vertical FTJ synapse array. Both the upper and lower Hf_0.5_Zr_0.5_O_2_ layers formed on a pillar showed the remnant polarization close to 16 μC/cm^2^, confirming the ferroelectricity of the switching layer in the three-dimensional structure. Also, both the upper and lower FTJs exhibited hysteretic resistance-voltage (R-V) curves using 100-ns-long programming pulses, with an ON/OFF ratio of 5, and the resistance change of the FTJ was stable up to 1000 cycles.

Moreover, ferroelectric two-dimensional (2D) van der Waals materials are attractive for FTJ utilization due to their significantly lower surface roughness and uniform thickness resulting from their layered structure, despite the difficulty of large-area fabrication. The materials innovation facilitates the performance improvement of FTJs. Wu et al. employed 1-layer graphene (1LG) and Cr as electrodes and 4-nm-thick CuInP_2_S_6_ (CIPS) as a ferroelectric switching layer into an FTJ [105]. Using PFM and capacitance-voltage measurements, the researchers confirmed the room-temperature ferroelectricity of CIPS and found a remnant polarization of about 8 μC/cm^2^. The FTJ based on 2D materials exhibits a counter-clockwise hysteretic R-V curve, which was acquired by pulse writing followed by DC resistance reading with 0.4 V bias. Therefore, hysteretic R-V curve indicates nonvolatile resistance change of the FTJ since the potential drop on the device went to zero between the writing and reading operation. Surprisingly, the TER reached as high as 10^7^, which is much higher than the TER of FTJs based on HfO_2_ and even an order of magnitude higher than that of perovskite FTJs. The significant TER was attributed to the semi-metallic property of 1LG, of which quantum capacitance near the Dirac point is low. When the polarization in the ferroelectric layer headed toward the 1LG, the charges were doped into graphene, and the Fermi level shifted upward. The Fermi level increase led the average barrier to decrease, inducing a larger tunneling current. The amount of the Fermi level shift was 1 eV in conjunction with Raman spectroscopy and Kelvin probe force microscopy, revealing the origin of large TER.

It should be noted here that in terms of performance (e.g., TER ratio), better performance with the highest TER (> 20,000%) for (Hf, Zr)O_2_ has been reported [102]. Nevertheless, the efforts presented in Sect. 3.4 deserve representative examples with regard to understanding of mechanism, integration density, material selection.

### Phase transition-based memristors

The principle of this type of filament-free switching memristors is attributed to the phase transition near the electrode. The details of the switching mechanism can be referred to in Sect. 2.5. Zhu et al. demonstrated memristive behavior from the lithiated MoS_2_ layer (Fig. 3d) [119]. The lateral MoS_2_ memristor with Au electrode exhibits reversible phase transitions from 2H to 1T phase and vice versa due to Li^+^ ions migration under the electric field. As Li intercalates between MoS_2_ layers, the semiconducting 2H phase, favored in multilayered MoS_2_, transforms into the metallic 1T phase, increasing conductance. The intercalated Li transfers electrons to Mo 4*d* orbitals, destabilizing the 2H crystal structure. The valence states change of Mo 4*d* orbitals was affirmed through X-ray photoelectron spectroscopy. While only characteristic Mo^4+^ peaks (at 228.8 eV for Mo^4+^ 3*d*_5/2_ and 232.1 eV for Mo^4+^ 3*d*_3/2_) appeared in the spectra for pristine MoS_2_, the spectra for lithiated MoS_2_ could be deconvoluted into peaks of Mo^4+^ and Mo^3+^ (at 228.1 eV for Mo^3+^ 3*d*_5/2_ and 231.3 eV for Mo^3+^ 3*d*_3/2_). Following the observation of chemical changes, Raman spectroscopy was utilized to confirm structural changes as well. The peaks at 384 cm^−1^ and 404 cm^−1^, observed in both samples, corresponded to the in-plane E_2g_^1^ and out-of-plane A_1g_ vibration mode of MoS_2_. For the lithiated MoS_2_, the characteristic Raman peaks of 1T-MoS_2_ (at 200, 225, and 355 cm^−1^) were also observed, revealing the existence of the 1T phase. For the electrical characterization, electrode A was biased, while electrode B was grounded (Fig. 3e). Applying the first negative bias on electrode A switched the device from the initial low resistance state to high resistance state, due to Li^+^ ion depletion near electrode B (Fig. 3f). After the forming-like process, the device was alternatively switched to the ON state and OFF state by applying positive and negative bias, respectively. The researchers emulated synaptic competition from the connected devices due to the limited Li^+^ ion supply in the system, and synaptic cooperation as well. The specific operation of synaptic application will be discussed later.

In addition, some materials, such as SrFeO_x_ (SFO) and SrCoO_x_, exhibit the topotatic phase transition between insulating brownmillerite (BM) and conducting perovskite (PV). Most of these materials manifest filamentary switching behavior due to accelerated oxygen ion migration from local Joule heating and oxygen vacancy channel in BM structure. Meanwhile, Rao et al. reported electroforming- and filament-free memristor based on epitaxial SFO oxide [125]. 55-nm-thick SrRuO_3_ (SRO) film, a buffer layer as well as the bottom electrode, was epitaxially grown on (001)-oriented SrTiO_3_ substrate followed by subsequent growth of 70-nm-thick SFO film by pulsed laser deposition. As a result of the deposition and in-situ annealing, PV-SFO and BM-SFO heterostructure was successfully formed. X-ray diffraction and TEM analysis on PV-SFO/BM-SFO heterostructure revealed that the thin and continuous BM-SFO layer was formed between the PV-SFO and the SRO layer, as well as the long-range ordering of the oxygen vacancy channel in parallel with the surface. The Au/PV-SFO/BM-SFO/SRO heterostructure exhibited memristive switching behavior without an additional forming process. Applying positive (negative) bias on the Au electrode, the device resistance decreased (increased) because of the oxygen ion migration-mediated phase transition. Oxygen ions were attracted toward the PV-SFO layer under the positive bias, and conducting PV-SFO near the PV-SFO/BM-SFO interface was transformed into insulating BM-SFO, increasing the resistance. Conductive AFM and area-dependent resistance confirmed interface-type filament-free memristive switching features consistent with the TEM results.

### Filling-controlled Mott transition-based memristors

The resistance switching of this type of memrisotrs is associated with the filling-controlled Mott transition, and the mechanism of the transition has already been covered in Sect. 2.6. Wang et al. reported resistive switching phenomena in rare earth perovskite nickelates, which have Mott physics [141]. The p-type GdNiO_3−x_ (GNO) films were epitaxially grown on Nb:SrTiO_3_ (NSTO) substrate with varying oxygen pressure during the deposition (100, 20, 2, and 0.2 mtorr, labeled as D1, D2, D3, and D4, respectively). Pt top electrode was biased, while NSTO was grounded. Figure 3g shows TEM images of D1 and D4, which exhibited high and poor crystallinity due to different oxygen deficiencies. DC-IV sweeps on the devices displayed distinctive features, as shown in Fig. 3h. While D1 exhibited negligible hysteresis in the IV curve, significant resistance switching was manifested in D4. A large ON/OFF ratio reached as high as 10^5^, accompanying the capacitance change, which could be occurred by depletion width change at the p-n junction (p-type GNO and n-type NSTO junction). The data retention (extrapolated 10^3^ > ON/OFF ratio for 10 years) and endurance (no significant change up to 10,000 cycles) test suggested that the device showed decent non-volatile memory characteristics. The left panel in Fig. 3i describes the resistance switching behavior of the heterostructure. The oxygen vacancies were migrated from GNO to NSTO under the positive bias, inducing the reduction of *E*_*g*_ and *E*_*F*_ in the GNO layer, as illustrated in the right panel of Fig. 3i (*E*_*g*_ and *E*_*F*_ of D1 to D4, which are GNO film with different oxygen vacancy concentration, were calculated using Tauc plot and measured by UV photoelectron spectroscopy). Therefore, the potential barrier for electron or hole injection and depletion width at the junction were reduced, resulting in the low resistance state and the high capacitance state. Conversely, a negative bias increases the concentration of oxygen vacancies in the GNO layer, leading to potential barrier recovery. A systematic TEM electron energy loss spectroscopy for the GNO/NSTO junction in the ON and OFF states revealed that the valence state of Ni was changed, consistent with the direction of *V*_*O*_ migration in Fig. 3i.

Moreover, the filling-controlled Mott transition of perovskite nickelates can be derived by proton doping as well as *V*_*O*_. Ramadoss et al. demonstrated SmNiO_3_ (SNO) and NdNiO_3_ (NNO) memristors, of which resistance was modulated by proton migration [139]. The SNO and NNO films are RF-sputtered on LaAlO_3_ substrates at room temperature, followed by annealing. The X-ray diffraction and temperature-dependent resistivity test confirmed that the film was crystallized into the target phase. The symmetric (both 100-nm-thick Pd) or asymmetric (100-nm-thick Pd and 10nm Ti/100 nm Au) electrodes were patterned on the nickelate channel to form planar devices. The nickelates near the Pd electrode were doped by proton through catalytic hydrogen doping under forming gas annealing at 100℃. After doping of proton in the nickelates, the resistance dramatically increased, indicating bandgap increase due to localization upon half-filling in Ni^2+^. The device with sub-micron channel length exhibited an ON/OFF ratio of 920 by applying 100-ns-long ± 5 V pulses. The ON/OFF ratio was a function of pulse amplitude and duration. The researchers also studied multi-level operation by varying the pulse amplitude.

Very recently, Wang et al. reported voltage-driven and *V*_*O*_ doping-controlled Mott transition in atomic-layer-deposited La_2_Ti_2_O_7−x_ (LTO) film on TiN substrate [142]. The mixed valence states of Ti ion (Ti^4+^ and Ti^3+^) and the composition of La versus Ti (1.07) in the pristine LTO film were confirmed by X-ray photoelectron spectroscopy. X-ray diffraction revealed that the crystal structure of the LTO film was monoclinic perovskite La_2_Ti_2_O_7_ crystalline phase, despite of tiny nonstoichiometry between La and Ti. The IV sweep for LTO film showed formingless bipolar resistance switching, starting with the low resistance state. The device was turned off (on) with the positive (negative) bias on the top electrode, which is W probe tip. The scanning photoelectron spectromicroscopy image on the LTO suface, which was attained by spatially mapping the intensity of O 1s peak in X-ray photoelectron spectrum, displayed the multiple OFF state region surrounded by the ON state matrix. The focused XPS on the region of interest, i.e. ON and OFF state region, exhibited distinctive proportion of Ti^4+^ and Ti^3+^ as well as oxygen vacancy. The Ti^3+^/Ti^4+^ ratio and oxygen vacancy/lattice oxygen in the ON state region is smaller than those in the OFF state region, suggesting the critical impact of electron filling in Ti 3d orbitals on the resistance state. DFT simulation regarding number of *V*_*O*_ in La_2_Ti_2_O_7_ lattice supported experimental data. The energy gap for the La_2_Ti_2_O_7_ incorporating two *V*_*O*_ was 0.47 eV, corresponding to the ON state. The energy gap increased as 0.75 eV by introducing one more *V*_*O*_ in the lattice, leading to the OFF state. Moreover, the researchers demonstrated that the electron correlation in LTO can be mitigated by nitrogen doping, resulting in decreased ON/OFF ratio.

## Computing applications with filament-free switching memristors

In this section, we present various examples of computing applications based on filament-free type of memristors. Recently, there has been increasing interest in the (non)volatile and dynamical switching characteristics of filament-free switching memristors because it can perform computations with a higher-complexity as well as the simple information storage. In this sense, an artificial synapse (Sect. 4.1)/neuron (Sect. 4.2), reservoir computing (Sect. 4.3), selector (Sect. 4.4), and self-rectifying memory device (Sect. 4.5) are sequentially described. Table 3 summarizes diverse demonstrations of computing applications based on filament-free switching memristors, which could help achieve a better understanding of their current state in computing.

**Table 3:** A summary table of various computing applications based on filament-free switching memristors

Application	Junction structure	Main mechanism	Array	(Local) circuit complexity	Specific functions	Targeted task	Pros/Cons	Refs.
Artificial synapses	Planar device Li_x_Mos_2_ with Au contacts	Phase transition	–	–	Synaptic plasticity and competition/cooperation	Bio-realistic functionality	Bio-realistic/planar device (low-scalable)	[119]
	Pt/Ta_2_O_5_/Nb_2_O_5−x_/Al_2_O_3−y_/Ti	Electron trapping/detrapping	32 × 32	–	Long-term potentiation and depression	MNIST dataset recognition	Uniformity/High operation voltage	[90]
	Ag/BaTiO_3_/Nb:SrTiO_3_	Ferroelectric polarization	–	–	Spike timing dependent plasticity	–	Ultrafast operation/CMOS incompatible (PLD)	[106]
	PT/α-MoO_3_/SrCoO_2.5_/Nb:SrTiO_3_	Phase transition	–	–	Long-term potentiation and depression	MNIST dataset recognition	Linear IV relationship/Large temperature dependency	[126]
Artificial neurons	Pt/anodized TiO_x_/Ti	Ion migration (*V*_O_)	20 × 20	A resistor and a capacitor	Short-term memory, self-rectification, LIF	AMP sequence generation	Uniformity/Small ON/OFF ratio	[57]
	Au/MAPbI_3_/ITO	Ion migration (*V*_I_)	2 × 2	1 memristor, 3 resistors, 1 capacitor, 2 potentiometers, 1 comparator, 1 pulse generator	Short-term memory, LIF	SNN demonstration	Low operation voltage/CMOS incompatible (halide perovskite)	[59]
	W/Pr_0.7_Ca_0.3_MnO_3_/Pt	Filling-controlled Mott transition	16 × 3 (simulation)	1 memristor with an external measurement and pulse input system	LIF	Fisher’s iris dataset classification	Tunable firing frequency/Insufficient physical demonstration	[153]
	W/WO_3_/PEDOT:PSS/Pt	Ion migration (proton)	–	2 memristors, 5 resistors, 1 capacitor, 3 potentiometers, 1 comparator, 1 timer	LIF	–	Highly bio-realistic HH neuronal dynamics/Circuit complexity	[155]
Reservoir computing	Au/WO_x_/W	Ion migration (*V*_O_)	(88 out of) 32 × 32	Physical reservoir measured by PCB	Short-term memory, memristor dynamics	MNIST dataset classification, second-order nonlinear prediction, spoken-digit recognition, Mackey–glass prediction	Uniformity/Slow resistance change	[30, 162]
	Ti/TiO_x_/TaO_y_/Pt	Ion migration (*V*_O_)	–	Physical reservoir measured by PCB	Short-term memory, memristor dynamics	Waveform classification, spoken-digit recognition, time-series prediction	Uniformity/Small dynamic current change	[31]
	Planar device SnS with Cr contacts	Electron trapping/detrapping	5 × 1	Physical reservoir	Optoelectronic signal, memristor dynamics	Korean characters and sentence recognition	Ability of optoelectronic signal processing/Small dynamic current change	[23]
Selector	Pt/CoO/ITO	Electron trapping/detrapping	–	1 selector(memristor) with wire-connected resistive switching memory	Threshold switching	–	Uniformity/Slow operation speed	[93]
Self-rectifying memristor	Pt/TaO_y_/nanoporous TaO_x_/Ta	Ion migration (*V*_O_)	16 × 16	–	Self-rectification, long-term potentiation and depression	MNIST dataset recognition	Uniformity/High operation voltage	[63]
	Pt/Al_2_O_3_/HfO_2_/Ti	Electron trapping/detrapping	64 × 64	–	Self-rectification, long-term potentiation and depression	MNIST dataset recognition	Uniformity/Low programmability	[182]
	Ru/Hf_0.8_Si_0.2_O_2_/Al_2_O_3_/Hf_0.5_Si_0.5_O_2_/TiN	Ion migration (*V*_O_)	30 × 30	–	Self-rectification, long-term potentiation and depression	Vector–Matrix Multiplication	Uniformity/Small ON/OFF ratio	[183]
	Pd/HfO_2_/WO_x_/W	Ion migration (*V*_O_)	–	–	Self-rectification, long-term memory	–	Uniformity/High operation voltage	[184]

### Artificial synapses

**Fig. 4:**
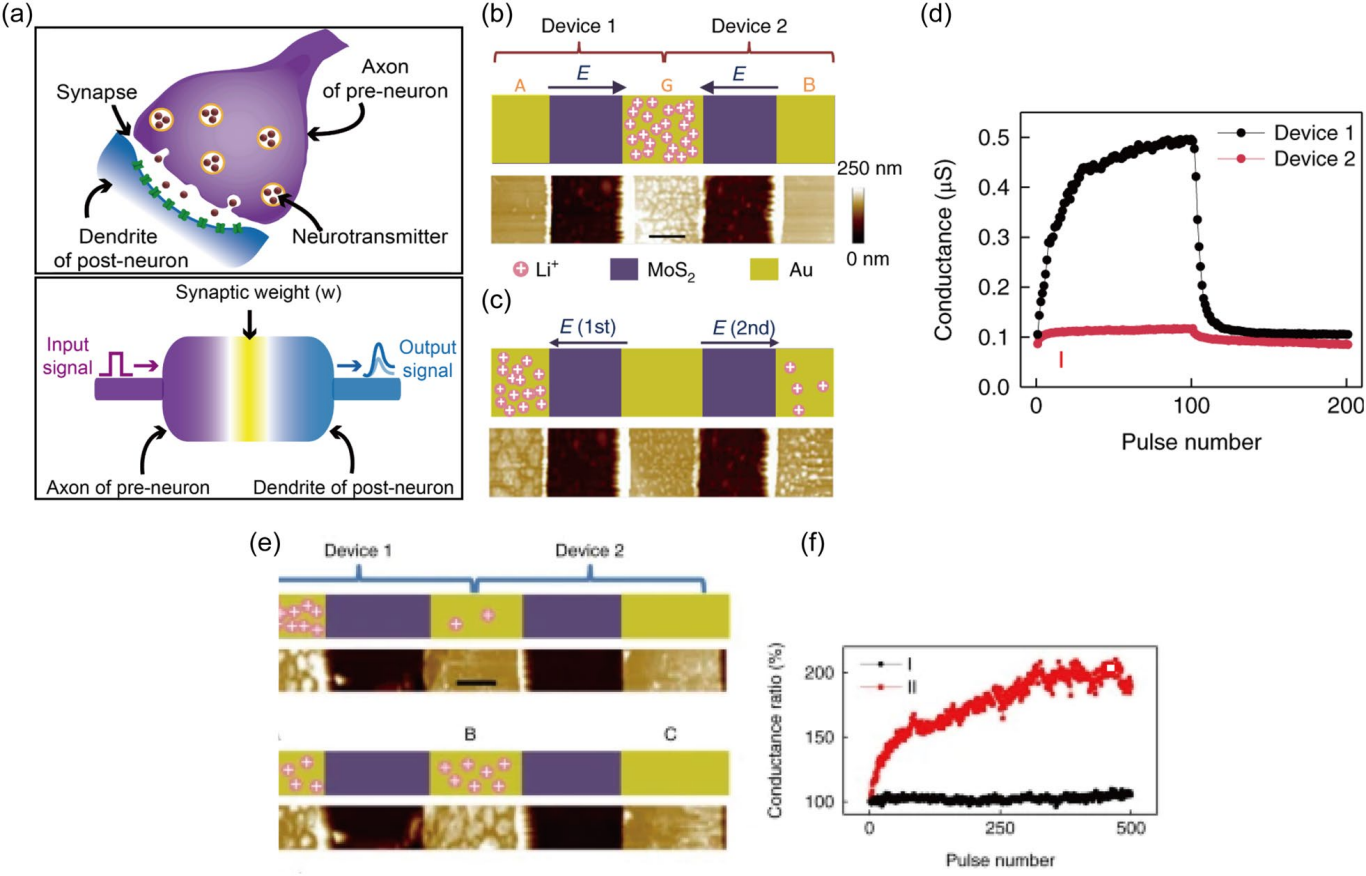
Artificial synapses and their synaptic functionalities. **a** Schematic illustration of biological synapses and the role of synaptic weight during the signal transmission. Reproduced with permission from Ref. [15]. Copyright 2020, John Wiley Sons. **b** The back-to-back memristors demonstrating synaptic competition and cooperation effect. **c** Different Li^+^ ion amount under the electrode of the memristors potentiated sequentially. **d** Synaptic competition effect between adjacent artificial synapses. **e** Li^+^ ion distribution for the artificial synapses exhibiting synaptic cooperation. **f** Distinctive conductance change of the device2 for the different Li^+^ ion configuration. Reproduced with permission from Ref. [119]. Copyright 2019, Springer Nature

As previously discussed, the neuron is a fundamental component of the biological nervous system, responsible for receiving signals from the dendrite, integrating and firing signals from the soma, and transmitting signals within the neuron through the axon. For information to propagate throughout the entire nervous system, neuronal signals must be transmitted between neurons through synapses formed between the axon terminals of pre-neurons to the dendrites of post-neurons. In the synapse, which is the connection of neurons, electrical signals known as action potentials are converted into chemical signals, i.e., neurotransmitters as shown in upper panel in Fig. 4a [143]. When an action potential reaches the axon terminal, synaptic vesicles release neurotransmitters into the synapse. These neurotransmitters diffuse across the synapse and bind to receptors on the dendrite, thereby exciting or inhibiting post-neuron activity depending on the type of receptor involved. The synapse plays a critical role not only in signal transmission but also in modulating the connectivity between neurons [144, 145]. Through a process known as synaptic plasticity as shown in the lower panel in Fig. 4a, the synapse can be strengthened or weakened by altering the population of receptors in response to neuronal activity. This ability to modify neuronal interconnections is considered a fundamental mechanism underlying learning and memory in the brain. Furthermore, synaptic interactions such as competition and cooperation between adjacent synapses are crucial for stabilizing neural networks [146]. Therefore, it is crucial to emulate various synaptic functionalities in order to demonstrate a biologically realistic neuromorphic system. Meanwhile, in the context of hardware-based artificial neural networks, the term "artificial synapse" typically refers to a key component of memristive computing that enhances the performance of artificial neural networks through crossbar structures [14, 15, 147, 148]. In a memristive neural network, an array of memristors is used, with the conductance of each memristor representing the synaptic weight of the corresponding connection in the artificial neural network. The artificial synapse, implemented by a memristor, enables efficient parallel and analog computation of vector-matrix multiplications, offering significant improvements in energy efficiency and processing time. To ensure precise operation of the memristive neural network, the conductance of each artificial synapse must be carefully programmed and exhibit sufficient non-volatility.

Zhu et al. demonstrated versatile synaptic functionality harnessing the Li^+^ ion modulation in MoS_2_ films [119]. The synaptic plasticity was readily emulated by the filament-free memristive behavior of their devices. As already discussed, Li^+^ ions drift across the MoS_2_ layer in response to external electric bias, resulting in conductance changes. Furthermore, the high diffusivity of Li^+^ ions in MoS_2_ films was utilized for effective ionic coupling, mimicking plasticity-related protein exchange in biological synapses and enabling biorealistic synaptic competition and cooperation effects. Figure 4b shows that the memristors are in a back-to-back connection, which means that two memristors share their ground electrode while their bias electrodes are separately operated. Depending on the sequence of events, if one synapse is excited first, the rest of the connected synapse cannot activate when an external bias is applied. Li^+^ ions are drifted toward the MoS_2_ layer in the memristor excited first, increasing the conductance. Since the Li^+^ ions are already drained out to the memristor excited first (Fig. 4c), even if electric bias is applied on the other memristor, the conductance does not change much due to the lack of Li^+^ ion that is needed to facilitate the semiconducting to metallic transition in the memristor (Fig. 4d and e). The competitive Li+ ion draining in limited Li+ ion supply conditions enables certain biorealistic synaptic functionality, namely the synaptic competition effect. In addition, synaptic cooperation was also demonstrated by preconditioning the back-to-back memristors (Fig. 4e). When the majority of Li^+^ ions are accumulated under electrode A (bias electrode for device 1), device 2 cannot be potentiated. On the contrary, after device 1 is potentiated by applying strong stimulation pulses, Li^+^ ions diffuse out from electrode A and are accumulated under electrode B (sharing electrode for both devices). Hence a sufficient amount of Li^+^ ions are supplied from electrode B to the MoS_2_ layer in device 2, resulting in the successful potentiation of device 2 (Fig. 4f). The synaptic cooperation effect of artificial synapses was expanded to a device network that consists of four memristors sharing a common electrode. As the number of potentiated neighboring memristors increases, the conductance of the non-stimulated memristor increases due to Li^+^ ion accumulation under the common electrode. Bio-realistic artificial synapses, as well as artificial synapses for memristive computing applications, have been reported elsewhere. Kim et al. implemented a 32 × 32 crossbar array of self-rectifying analog charge trap memristor based on a Pt/Ta_2_O_5_/Nb_2_O_5−x_/Al_2_O_3−y_/Ti. The accuracy of the MNIST test based on their experimental synaptic characteristics reaches as high as 91%, indicating that the improved retention by employing blocking layers for charge loss enables the memristor to work as the artificial synapse [90]. In addition, Ma et al. demonstrated the Ag/BaTiO_3_/Nb:SrTiO_3_ structured ferroelectric memristor and simulated the memristor-based artificial neural network, which exhibits over 90% accuracy [106]. A relatively stable and reliable ferroelectric effect could result in a high-performance artificial synapse based on a filament-free memristor.

### Artificial neurons

**Fig. 5:**
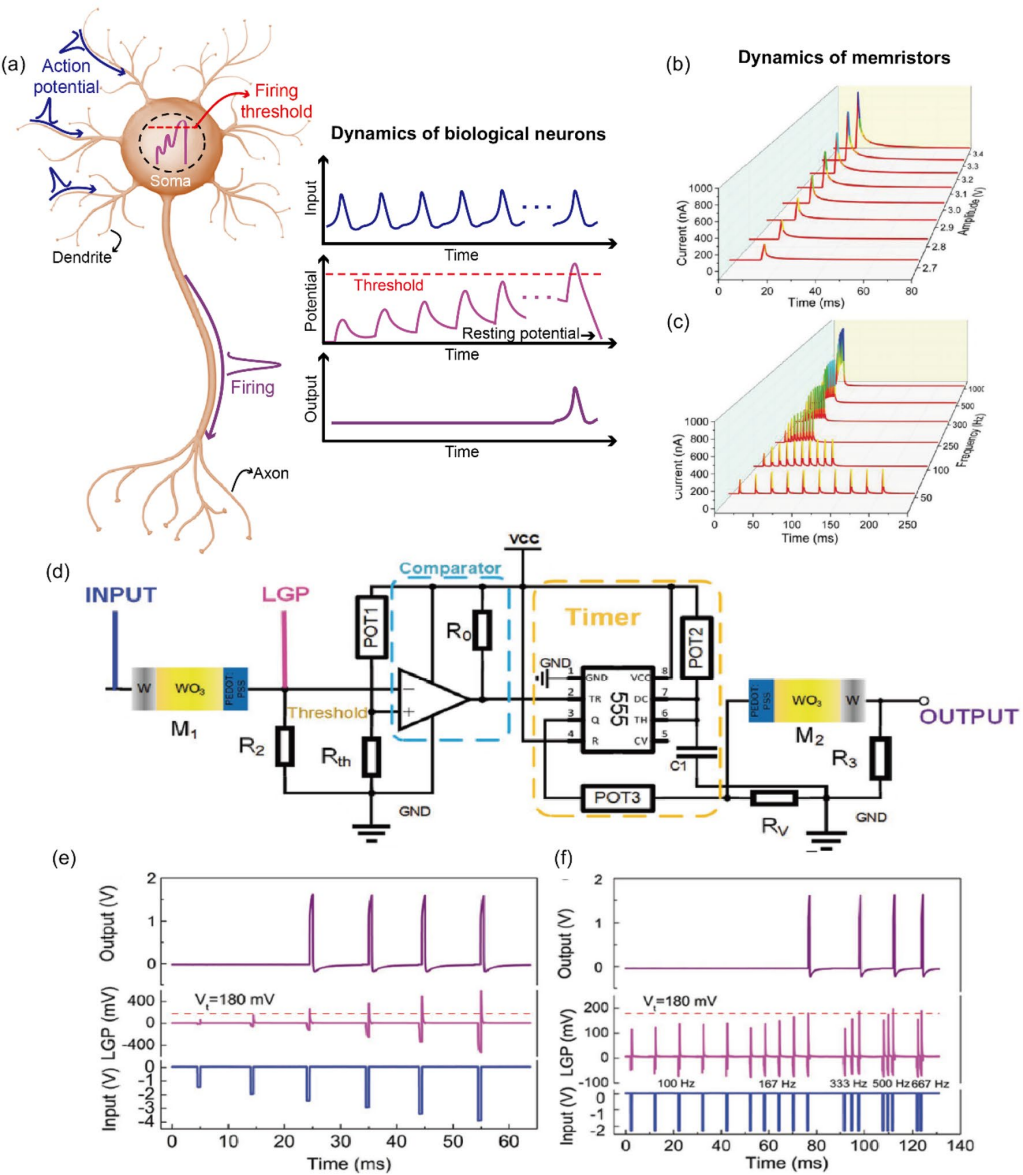
Artificial neurons and their neuronal functionalities. **a** Schematic illustrations of biological neurons consisting of dendrite, soma, and axon (left) and the representative integrate-and-fire dynamics of the neurons (right). Reproduced with permission from Ref. [156]. Copyright 2022, John Wiley and Sons **b**, **c** Neuronal dynamics emulated by the filament-free memristor as a function of amplitude (**b**) and frequency (**c**). **d** A schematic circuit diagram of the quasi HH artificial neuron based on two non-filamentary memristors. **e**, **f** Plots of the electrical neuronal dynamics using the designed quasi HH neuron as functions of amplitude (**e**) and frequency (**f**). Reproduced with permission from Ref. [155]. Copyright 2019, John Wiley and Sons

In the human brain, biological neurons, which are composed of dendrites, soma, and axons, are crucial for processing information and interacting with other neurons by receiving, integrating, and transmitting neural signals in the form of electrical spikes (referred to as action potentials) (Fig. 5a) [149, 150] . As shown in Fig. 5a, when the dendrites of a neuron receive action potentials (represented as blue line) from other pre-neurons, an electrical activation of the neuron occurs. As the number of incoming action potentials increases, the membrane potential (represented as light purple) can be integrated until it exceeds a certain threshold (represented as red). Once the membrane potential is over the threshold, the neuron fires and transmits an action potential to the post-neurons via the axons (represented as violet). After that, the neuron spontaneously returns to its resting potential. These dynamics of biological neurons are considered as a fundamental principle of signal processing in the brain [149, 150]. Similarly, memristors can mimic the integrate-and-fire dynamics of the biological neurons via the voltage pulses [57, 59, 151–156]. Notably, filament-free memristors generally feature the temporal information processing and volatile switching characteristics based on different internal state variables such as concentration of ions, temperature, built-in electric field, and interfacial states, thereby emulating the essential dynamics of the biological counterpart [57, 59, 151–155]. Furthermore, when combined with external electrical components or circuitries, a wide range of neuronal functionalities (23 types of known biological neuron spiking behaviors) are allowable [151]. Note that the dendrites and axons of biological neurons can correspond to the top and bottom electrodes (or vice versa) while the soma can be regarded as a switching layer.

For example, Huang et al demonstrated a quasi Hodgkin-Huxley (HH) artificial neuron by employing W/WO_3_/PEDOT:PSS/Pt junction structure [155]. The switching principle was attributed to the phase transition between conducting H_x_WO_3_ and semiconducting WO_3_. Note that the HH neuron is a bio-realistic neuron model based on different ion channels and ionic motion in the biological neurons. As shown in Fig. 5b and c, current responses of the fabricated device were observed to increase according to the amplitude and frequency of input voltages. In addition to this, the increased current automatically decayed in a few milliseconds without the reverse input voltages. Figure 5d shows a circuit diagram of an HH artificial neuron with the fabricated device, where WO_3_/PEDOT:PSS memristors (*M*_*1*_ and *M*_*2*_) were employed for the main source of membrane potential and spiking generation. When input voltages were introduced into the suggested circuit (blue), the current response of *M*_*1*_ was converted into the voltage form by the wired resistor (*R*_*2*_), which was represented as LGP (local graded potential, light purple). Depending on the input amplitudes and frequencies, different LGPs were formed and then an output signal from the comparator (dotted blue box) could be generated when the LGP was higher than the threshold value. This subsequently caused the Timer (yellow dotted box) to induce a voltage pulse with a certain pulse width, which was applied into the *M*_*2*_ with a series of *R*_*3*_. Based on the principle of voltage division, the voltage applied across *R*_*3*_ could act as the firing signal (violet). Because the fabricated device had an internal electric field during the decaying period, the voltage decreased to negative value below zero, enabling the quasi HH neuron. Figure 5e and f show spatial and temporal integration-and-fire dynamics based on the suggested circuit, respectively. If many inputs simultaneously arrive at the neuron circuit, the amplitude of applied voltage could be effectively increased. As shown in Fig. 5e, the LGP was observed to increase as the amplitude of input voltage increased, thereby generating the output firing signals when the LGP value exceeded the threshold. Similarly, if the inputs reach the neuron at the different rates, the frequency of applied voltage becomes changeable. As shown in Fig. 5f, the LGP was observed to increase as the five input voltage pulses were introduced with different frequencies. As the frequency was increased, the LGP could overcome the threshold, which generated the output signals. Based on the suggested memristive neuron circuit, a quasi HH neuron with bio-realistic dynamics has been physically implemented successfully.

Further, artificial neurons have been proposed by employing diverse types of filament-free switching memristors. For example, Park et al demonstrated reliable leaky integrate-and-fire neuronal dynamics with an anodized TiO_x_ memristor in parallel with a capacitor [57]. Yang et al. also combined a CH_3_NH_3_PbI_3_ memristor with an external comparator and a pulse generator, suggesting it as an artificial neuron [59]. As a result, utilization of filament-free switching memristors can be regarded as one promising method to implement an artificial neuron embedded with biological dynamics and facilitate development of neuromorphic computing.

### Reservoir computing

**Fig. 6:**
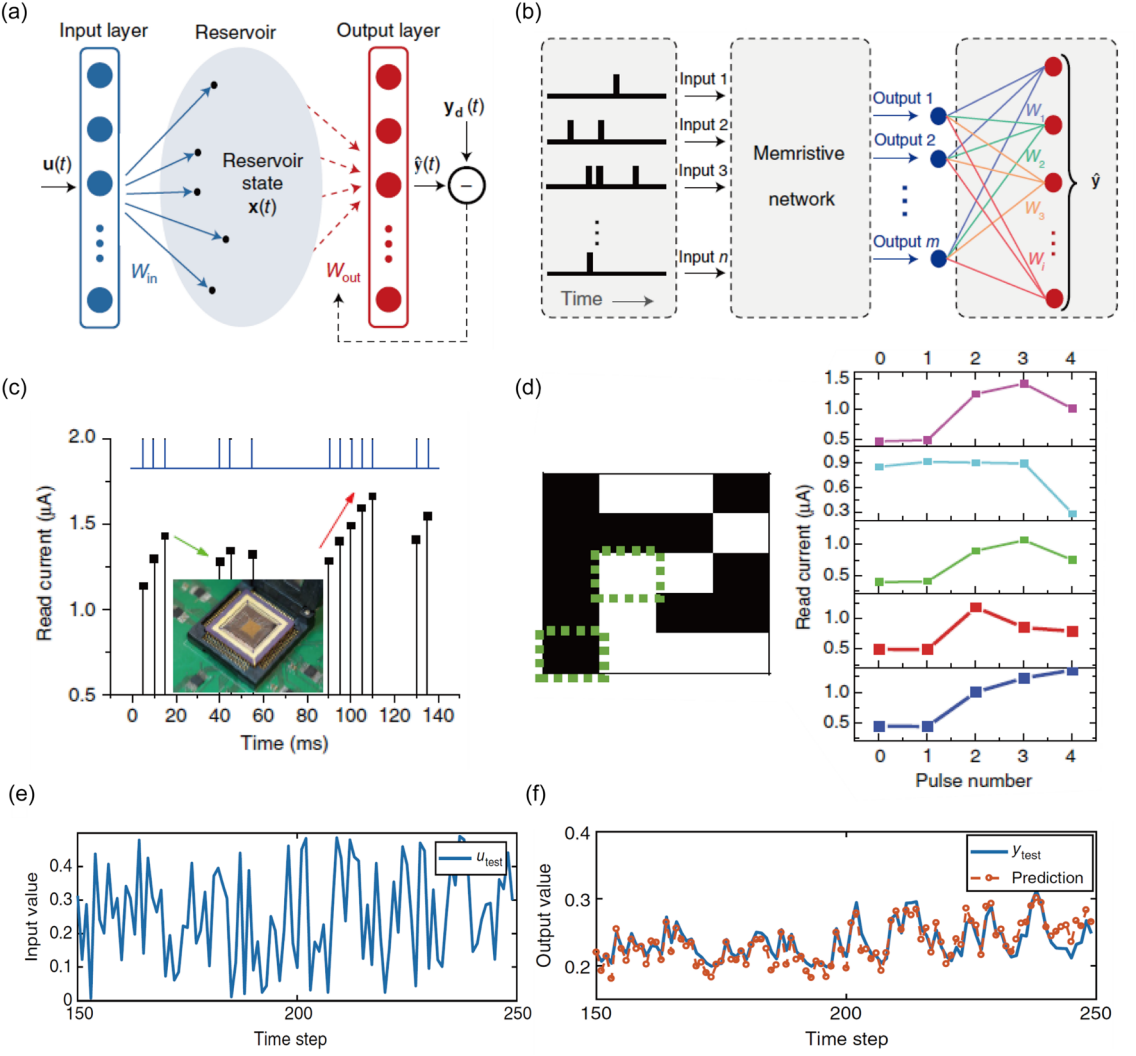
Reservoir computing. **a** A illustration of reservoir computing consisting of input layer, reservoir, and output layer. The reservoir can nonlinearly transform incoming temporal inputs into reservoir states in a new feature space, which are then further learned and analyzed at the output layer. **b** A hardware implementation scheme of reservoir computing based on filament-free switching memristors. Reproduced with permission from Ref. [164]. Copyright 2021, Springer Nature. **c** A plot of read currents (reservoir states) in response to programming voltage pulses with varying input timings. **d** A digit pattern (left) and the reservoir states through five memristors with respect to the five input pulse trains corresponding to each row of the digit pattern. **e**, **f** An input pattern of the second-order nonlinear problem (left) and its solving result with a memristive RC system (right). Reproduced with permission from Ref. [30]. Copyright 2017, Springer Nature

There has been increasing demand for efficient processing approach to address temporal information in many fields such as healthcare, smart systems and internet of things (IoT). Generally, non-volatile memristor-based artificial neural network can handle static information such as handwritten digit patterns in which an input image at a certain time is completely lost after it is fed to a neural network. In other words, it is hard to process time-series data with a sequential order because an input at a time step (t) is not stored when receiving an input at the next time step (t + 1). Although several approaches to temporal information processing have been suggested including backpropagation through time algorithm and recurrent neural networks [157, 158], these approaches require compute-intensive computations to correlate data between different time steps. In order to alleviate these problems, reservoir computing (RC) system has been proposed (Fig. 6a) [23, 30–32, 159–164]. As shown in Fig. 6a, a RC system typically consists of a reservoir and input/output layer. The reservoir is a set of nonlinear elements coupled with one another, which are volatile and recover to the initial state after each operation, indicating that there is no need to train this part during learning processes. Because only synapse weights located between reservoir and output layer are updated according to a simple single-layer learning algorithm, RC systems are regarded as a much more efficient method to address the temporal input data. It is known that there are two main properties for the RC system, namely, fading memory and separation [159, 160]. Considering that the filament-free type of memristors is able to nonlinearly transform from voltage inputs to current outputs with a short-term memory effect, it can efficiently implement the physical reservoir states without complex loops from multiple nodes in the software-based RC systems [23, 30–32, 162]. Hence, the most compute-intensive phase in RC systems, which operate a reservoir for each time step, can be effectively simplified with the memristor-based physical reservoir. Figure 6b shows a schematic diagram of a RC system with memristors. As shown in Fig. 6b, when time-dependent voltage pulses are introduced into the memristor-based physical reservoir, reservoir states in the form of current output are then processed and analyzed in the output layer in which a classification or regression is generally employed for the synaptic weight updates.

For example, Du et al utilized a memristive crossbar array combined with a custom-designed PCB board for a RC system [30]. Figure 6c and d show the temporal information processing ability of the fabricated memristive array in response to certain temporal orderings of the voltage pulse inputs. For example, as shown in Fig 6c, current responses decayed unless the subsequent voltage pulse was applied into the memristor at a short interval (green arrow). However, the current became high when several voltage pulses were applied to the memristor at a short interval (red arrow). This result indicates that dynamic memristors with a short-term memory effect can present different current outputs (i.e., reservoir states) when receiving different voltage pulse inputs. Similarly, a digit 2 image that had black and white pixels with different orders at each row could be converted into five sequences of voltage pulses (Fig. 6d). Because the relative timing of the programming pulses varied, the memristor could generate different reservoir states at each row of the image, as shown in Fig. 6d. The resultant reservoir states were fed into the output layer for the learning process to allow the RC system to recognize the image as digit 2. With these characteristics, classification of handwritten digit images has been successfully performed by using the fabricated crossbar array, which achieved a recognition accuracy of 88.1 % when the reservoir consisted of only 88 memristors. Further, a second-order nonlinear equation was experimentally addressed by using the memristor-based RC system (Fig. 6e and f). Because the used equation was dependent on the recent past outputs but not on the far past, this type of problem was selected for the memristor-based RC system test. As shown in Fig. 6f, prediction results obtained from the memristive RC system after the learning process (red circles) were comparable to the theoretical outputs (blue line) when certain inputs were introduced into the equation. The normalized mean squared error between two results was found to be 3.13 × 10^−3^, verifying that the memristive RC system was capable of processing time-dependent information.

In addition to this, there have been also other demonstrations regarding physical RC systems based on filament-free switching memristors. Zhong et al. utilized TiO_*x*_/TaO_*x*_ bilayer memristor with multiplexing masks as a physical reservoir, which performed spoken-digit recognition of 97.6 % accuracy and henon-map prediction with 0.046 normalized mean square error [31]. Moon et al. demonstrated a WO_*x*_ memristive crossbar array mounted on the test board system and they exhibited the energy efficiency of the suggested memristor-based RC system when compared with CPU- and FPGA-based RC systems [162]. Consequently, these efforts suggest the availability of filament-free switching memristors to a physical reservoir in RC hardware systems that can be potentially utilized for efficient time information processing.

### Selector

Memristive crossbar arrays have the strong potential to become a mainstay for future memory and artificial neural network technologies. However, when memristors are arranged into a crossbar array structure, there is a significant problem called a crosstalk issue [165, 166]. Because sneak currents flow through the unselected memristors in the array, incorrect programming and reading operations can cause the degradation of the computing performances in terms of reliability, energy, and time. To circumvent this issue, although adding a transistor on each memristor is a common solution, this approach can cause other problems due to the three-terminal structure and unstackability of transistors. Therefore, a two-terminal and 3D stackable selector in series with a memristor is a more attractive solution. Various types of selectors are suggested, such as a diode [167, 168], crested tunneling barrier [169–171], mixed ionic-electronic conductor [172, 173], insulator-metal transition [174–176], Ovonic threshold switch [177–179], and diffusive memristor [180, 181]. Except for the diode and the crested tunneling barrier, other selectors usually require an undesirable electroforming process to precondition the device in an appropriate operation mode, which indicates the filament-related conduction of those selectors. The diode selector is limited to nonpolar memristor, of which the resistance switching for low-to-high (RESET) and high-to-low (SET) resistance states occurs in the same polarity. The crested tunneling barrier suffers from a low ON current. Thus, the large scale memristive crossbar array demands a filament-free selector with high conductance and symmetric conduction behavior.

**Fig. 7:**
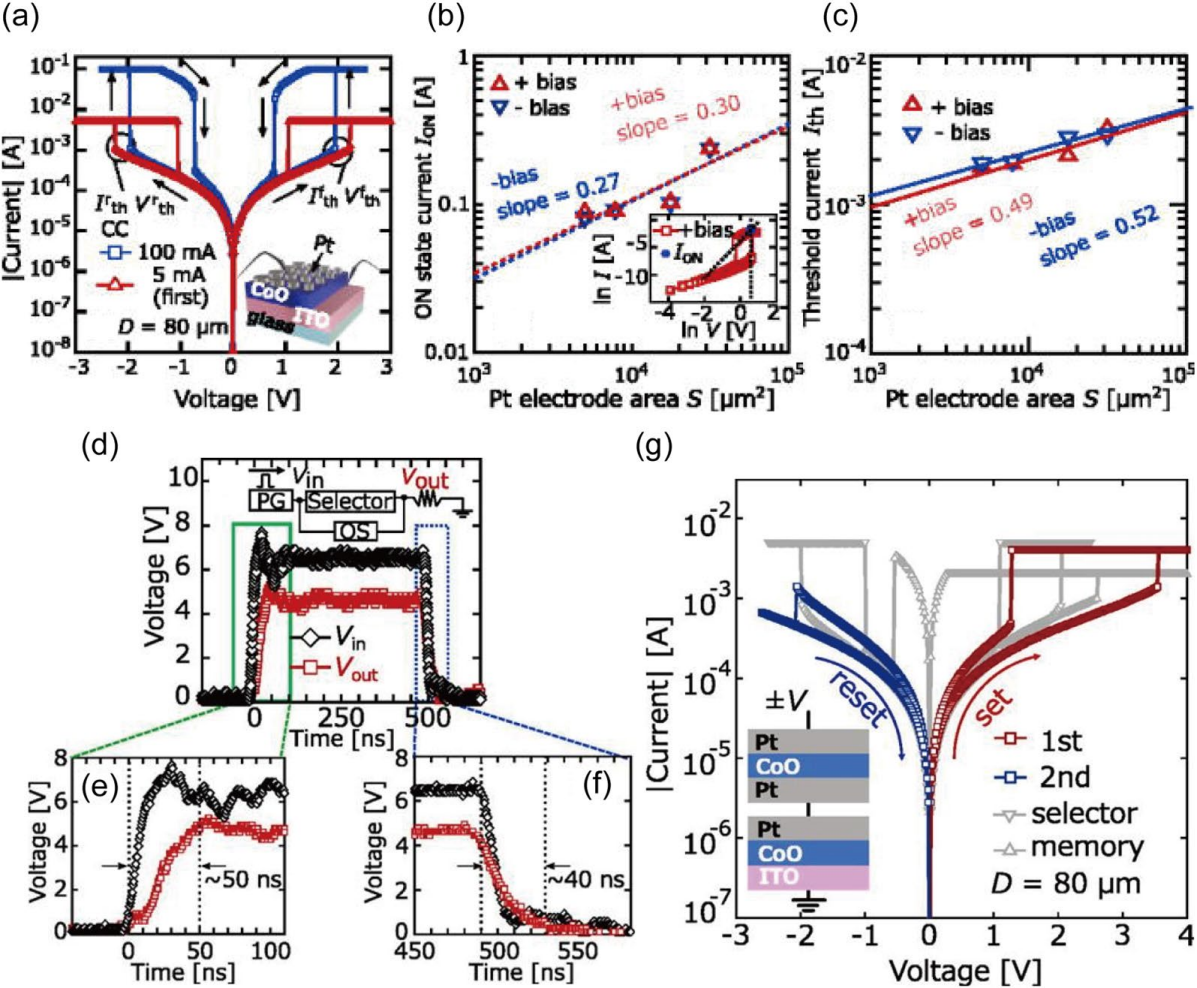
Selectors. **a** A representative *I-V* curves for the electron trapping-based memristor showing threshold switching. **b**, **c** The area dependency of the current in ON (**b**) and OFF (**c**) states. **d**–**f** The pulse measurement for the switching evaluation and enlarged data during the switch on (**e**) and off (**f**). **g** *I*-*V* curves for one-selector one-resistor cell showing successful resistance switching hysteresis. Reproduced with permission from Ref. [93]. Copyright 2020, AIP Publishing

Recently, Saitoh et al. developed a filament-free selector based on a volatile electron-trapping/de-trapping memristor [93]. The detailed conductance switching mechanism for the memristor is already discussed previously (in Sect. 3.3). As shown in Fig. 7a, the initial cycle exhibits a clear resistance switching behavior, suggesting a forming-free operation. Notably, the current abruptly increases in the forward direction (the absolute value of the voltage increase) and also abruptly decreases in the reverse direction (the absolute value of the voltage decrease), exhibiting a volatile memristive behavior that serves as a threshold switch. Note that the threshold switching event can be explained by the transition from trap-unfilled SCLC to trap-filled SCLC (see, Sect. 2.3 and 3.3). The currents for the ON and OFF states exhibited an area dependency of around 0.3 and 0.5 in the logarithmic plot, respectively (Fig. 7b and c). These results are consistent with the filament-free switching mechanism. This is because in filamentary types of memristors, ON current could almost maintain unless device area becomes smaller than the nanoscale filament. This unique area-dependency forecasts the excellent performance of the selector in the ultra-scaled dimension, for instance, the ON current density of around 500 MA/cm^2^ and the ON/OFF ratio over 10^4^ when the diameter shrinks to 20 nm. Also, the selector based on the electronic trapping effect is advantageous in minimizing the variation of the threshold voltage. The threshold voltage for both positive and negative polarities exhibits a relative standard variation of 0.02, much lower than that of other threshold switches. The transition speed of the selector was evaluated via the pulse measurement, as shown in Fig. 7d. The switch-on and -off times take 50 ns and 40 ns, respectively (Fig. 7e and f). The transition is moderate, considering that the resistance change comes from trap filling by electrons. In addition, the selector was integrated with the nonvolatile memristor, i.e., resistive switching memory (RSM), to construct the one selector—one resistor (1S1R) cell. Figure 7g shows successful SET and RESET operations of the memristor connected in series with the selector. At a low voltage regime, the resistance of the selector is larger than that of RSM, leading more significant voltage drop on the selector. As increasing the voltage positive, the resistance of the selector suddenly drops at the threshold voltage (~ 3.5 V), then most of the voltage is applied to RSM, and SET operation occurs. An increase in current through the 1S1R cell was observed in the low voltage region because the OFF resistance of the selector recovered while the resistance of the RSM remained low. As the voltage negatively increases, RESET operation occurs at around – 2 V. This is significantly higher than the reset voltage of the RSM itself (~ − 0.6 V) due to the voltage drop across the selector. One thing to note is that the RESET operation occurs independently of the selector operation. This might lead to undesirable write disturbance on unselected cells. Therefore, although the performance of the selector and its matching with the RSM need to be further improved, the selector based on filament-free memristors has shown some attractive properties, such as narrow distributions of their characteristics.

### Self-rectifying memristor

Memristive crossbar arrays have a strong potential to become a mainstay for future memory and artificial neural network technologies. However, as mentioned above, when memristors are arranged into crossbar array structure, the sneak current issue needs to be solved. A memristor embedded with a rectifying function, referred to as self-rectifying memristor, has attracted some attention because it can maintain a small footprint and the two-terminal structure without the having the voltage/current matching issue [57, 63, 89, 182–184]. In contrast to filamentary types, filament-free types can be readily engineered into a self-rectifying memristor because they do not have conductive filaments connected between two terminal electrodes. For example, a Schottky barrier can be designed for a rectifying function in the case of non-filamentary memristors. This method may not be applicable for filamentary memristors because two-terminal electrodes are connected via conductive filaments. Several self-rectifying memristors have been demonstrated based on non-filamentary memristors.

**Fig. 8:**
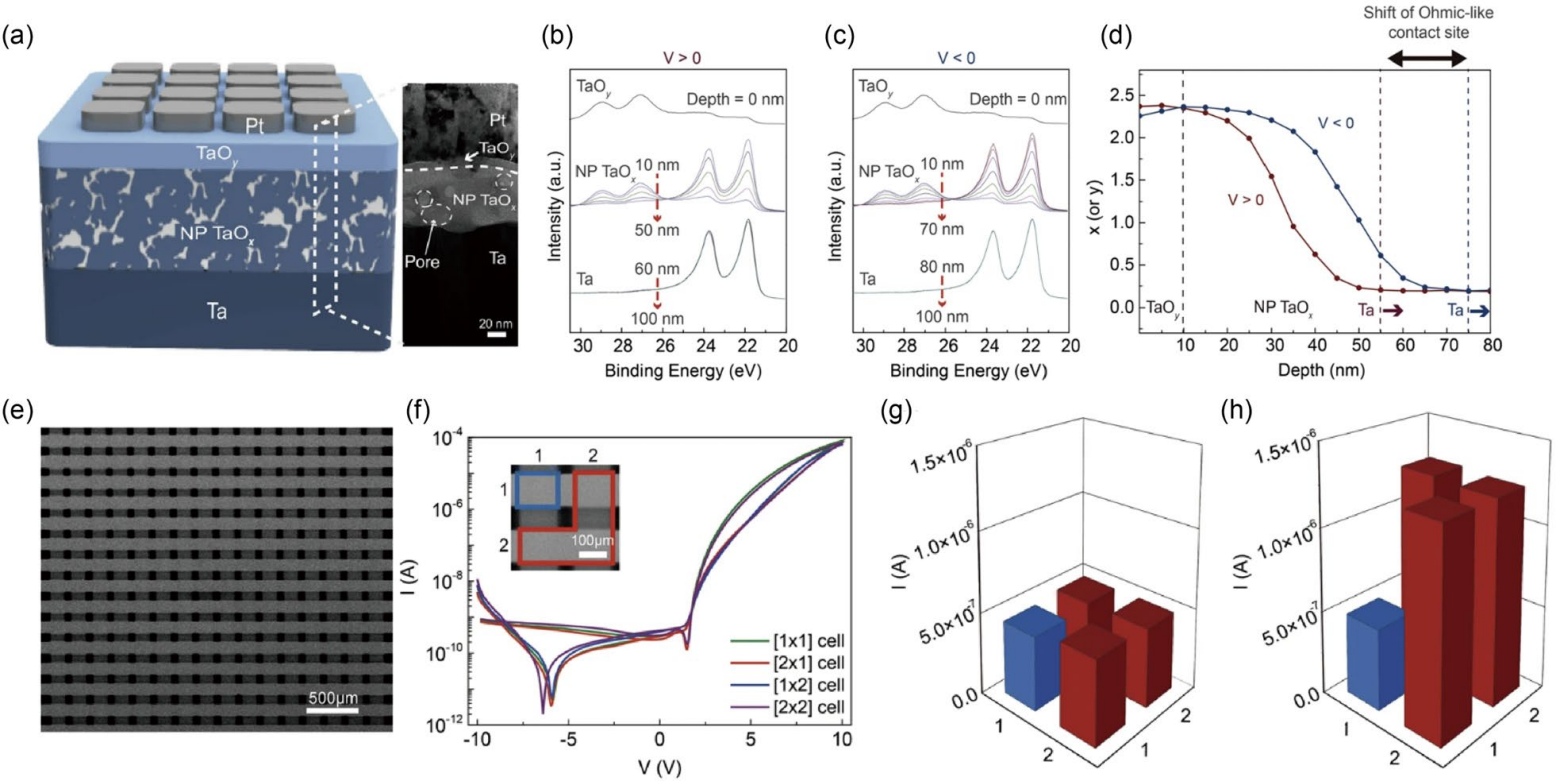
Self-rectifying memristors. **a** A schematic illustration of the NP TaO_*x*_ memristor and its cross-sectional TEM image. The white dotted circles indicate nanopores within the NP TaO_*x*_ layer. **b**–**d** XPS analysis results of the fabricated device as functions of the depth and the applied voltage. **e** A top-view SEM image of the crossbar array consisting of the NP TaO_*x*_ memristors. **f** *I*-*V* curves of four memristors in the 2 × 2 sub-array. **g**, **h** Histogram plots of the reading current values of the selected (blue) and unselected memristors (red) when the unselected memristors are in the OFF (**g**) and ON states (**h**). Reproduced with permission from Ref. [63]. Copyright 2018, Springer Nature

Recently, Choi et al. designed and fabricated a self-rectifying memristor consisting of Pt/TaO_*y*_/nanoporous (NP) TaO_*x*_/Ta junction structure (Fig. 8) [63]. Figure 8a shows a schematic diagram of a device structure and its corresponding cross-sectional TEM image, confirming that different nanopores (radius of ~ 10–25 nm) were located inside the NP TaO_*x*_ layer. To form the NP TaO_*x*_ layer, they have anodized the upper side of the bottom Ta electrode based on a sulfuric acid solution with HF and H_2_O. Owing to the top-bottom oxidation of the anodization process, *V*_*O*_ gradient varying with the depth was obtained, implying that *V*_*O*_ migration was responsible for the switching event. The top TaO_*y*_ layer was deposited to obtain a robust Schottky barrier at the Pt/TaO_*y*_ interface and prevent the Pt from penetrating through the nanopores of NP TaO_*x*_ layer. Figure 8b–d show ex-situ XPS analysis results and a plot of estimated stoichiometry *x* (or *y*) according to the depth as a function of applied bias polarity (V > 0 and V < 0). As shown in Fig. 8b–d, Schottky barrier and Ohmic-like barrier could be formed at Pt/TaO_*y*_ interface (*y* ~ 2.4) and NP TaO_*x*_/Ta interface (*x* ~ 0.2), respectively. Further, these analyses supported the bias-dependent *V*_*O*_ migration during the switching. As shown in Fig. 8d, when a positive bias was applied to the top Pt electrode (V > 0), positively charged *V*_O_ drifted toward the bottom Ta electrode, and the effective thickness of TaO_*x*_ could be decreased. This resulted in a higher conductance state (i.e., ON state). However, when a negative bias was applied to the top electrode (V < 0), the insulating thickness of TaO_*x*_ would be increased via the reverse mechanism, leading to a lower conductance state (i.e., OFF state). Because of these reasons, the fabricated device exhibited an asymmetric bipolar switching behavior without any forming process. The fabricated device was expanded into a 16 × 16 crossbar array to investigate the effect of self-rectifying property on the sneak currents as shown in Fig. 8e. After verifying the asymmetric bipolar switching curves of the selected 2 × 2 subarray (Fig. 8f), a crosstalk test was performed (Fig. 8g and h). In the subarray, the selected memristor ([1 × 1], blue box) in the OFF state could be correctly read as the OFF state regardless of whether neighboring memristors were all OFF (Fig. 8g) or ON states (Fig. 8h). Therefore, based on the self-rectifying memristive crossbar array, an artificial neural network for recognizing handwritten digit images has been simulated, showing a better accuracy by suppressing sneak currents.

As another example, Kim et al. demonstrated a self-rectifying Pt/NbO_*x*_/TiO_*y*_/NbO_*x*_/TiN memristor based on the trap-mediated switching event and an asymmetric energy band structure [89]. Jeon et al. designed a Ru/Hf_0.8_Si_0.2_O_2_/Al_2_O_3_/Hf_0.5_Si_0.5_O_2_/TiN junction structure where the Al_2_O_3_ layer limited the self-diffusion driven by the concentration gradient of *V*_*O*_ and improved the retention capability [183].

## Outlook and conclusion

Memristive computing systems have considerably advanced in recent years and are still being actively developed to realize their strong potential in computing technologies. As described in Sects. 2 and 3, filament-free memristors have been suggested based on various materials such as metal-oxide, perovskite, and 2D materials. They all have their own switching mechanism, but the switching characteristics are commonly determined by the physical and chemical phenomena across the whole switching layer and near/at the interface, which could be differentiated from filamentary memristors based on the formation and rupture of local filaments. Although the current filament-free memristors should be further developed and optimized to bring them into reality (Table 2), there is a high possibility that filament-free type of memristors could exhibit dynamic, uniform, and stable switching behaviors due to the intrinsic nature of filament-free switching (Introduction and Section 2). Such properties are highly desirable for numerous computing applications such as neuromorphic device/computation, time-correlated computing, future memory technologies. Further, more mature approaches based on non-filamentary memristors would likely find novel computing schemes beyond (or together with) the current memristive computing system. This is mainly because unexplored computing schemes so much typically involve the ability to process time-series data, such as spiking neural networks (SNNs) [185–187] and reservoir computing [3, 30–32, 159–164], which is inappropriate to many filamentary types of memristors with static switching behaviors. For example, the SNNs, which is a promising candidate as the next-generation neural network for neuromorphic computing, are operated based on the interval and timing of spikes [185–187]. These temporal computations may naturally call for time-correlated and dynamic switching characteristics to implement SNNs on memristive computing systems. The switching uniformity is also essential to correctly update various synaptic weights in a network with the same information of spiking timings. In other words, synaptic weights applied with the same spiking timing need to be updated with the same change amount; otherwise, the timing information can be disturbed. Moreover, these networks operate with many spikes (e.g., 60,000 pulses at each epoch for MNIST handwritten digits), requiring the switching stability. Although many of spiking artificial neurons discussed in Sect. 4.2 are still at the beginning of their development, filament-free switching memristors have shown promise for the futuristic SNN frameworks.

There are also remaining challenges of computing applications with non-filamentary memristors. To achieve realistic solutions, significant interdisciplinary efforts are required involving material and device, crossbar array, algorithm, and circuit. For example, many filament-free switching memristors, especially based on ion migration and electron trapping/detrapping process, suffer from the retention and switching speed issues, which makes them difficult to use for artificial synapse and memory applications. The structural engineering and band structure design is one of the promising strategies to mitigate short retention time. Choi et al. utilized nanoporous oxide structure as the switching layer to improve the retention capability (up to ~ 10^4^ s) [63]. This might be attributed to the large number of nanopores within the TaO_x_ switching layer by impeding the spontaneous dissipation of *V*_O_ (or charges). Similarly, Kim et al stabilized the trapped electrons through the formation of high interfacial barriers that limits the possibility of direct tunneling from trap to electrode, thus showing the acceptable retention property with 10^5^ s at 150 ℃ [90]. Another approach involves the stability of chemical interaction between ions and electrode. Solanki et al verified that the chemical reactivity of migrating iodine vacancies with the Ag electrode at the interface resulted in the memory effect with the long retention capability (250 h in an inert environment) [188]. Moreover, the switching speed is typically related to the switching kinetics. For example, Meyer et al investigated the electric field-driven nonlinear increase of the ion mobility in a bilayer oxide memristor [189]. As a higher electric field decreased potential barrier of ion transport, a relatively fast operation was obtained under the pulse-width range of 1–10 μs at a moderate voltage of 3 V. Similarly, dimensional scaling method is also one candidate for the lower operating time. As discussed in Sect. 2.2, lower-dimensional materials able to reduce the length of ion migration can be expected to enhance the switching speed. Further, based on a properly designed band structure with ferroelectric polarization, Ma et al. proposed a sub-nanosecond filament-free switching memristor [106]. A higher carrier concentration and a metal electrode with lower work function were observed to make the *Φ*_B_ and *W*_d_ smaller, thus achieving the operating speed as fast as 600 ps. Sneak current issue in the array can also be alleviated via operation schemes. Rao et al. reported a timing selection method that utilize a voltage-dependent delay time of volatile switching behaviors in the memristive selector and then successfully confirmed the suppression effect on the sneak current in the array [190]. At the algorithmic and circuit level, Moon et al suggested the concept of the virtual node to effectively capture time information by separately recording the current responses to time-dependent inputs at each time interval [162]. In addition to this, because a certain switching feature can concurrently act as either an advantage or a disadvantage depending on the type of computing application, it is prerequisite to figure out whether which characteristics are favorable for the desired computing application. For example, dynamic switching with short-term memory effect can be beneficial to temporal information processing. However, this volatility is not suitable for the conventional matrix-vector multiplication based on memrsitive crossbar arrays. Thus, a holistic approach can provide more effective route for the development of non-filamentary memristors and their computing applicability.

Meanwhile, CMOS compatibility is an important requirement for the utilization of memristor technology into the real-world device fabrication. For the perspectives of mass production and the device yield, some of the fabrication technologies and materials could be limited, for example, due to high-temperature deposition or annealing on back-end-on-line, too precious materials, and switching materials with high-diffusive species. Among them, one important consideration is the industry compatibility of the material systems. For example, material combination of industry-friendly electrodes (e.g., Ti, TiN and W) and switching layers (e.g., HfO_x_, TiO_x_, and TaO_x_) can be more facile for a factory production. However, using some perovskites (e.g., CH_3_NH_3_PbI_3_) vulnerable to heat and moisture as the switching layer could lead to the degradation of operating stability over time [191]*,* which needs to be further improved in terms of the CMOS-compatible fabrication. In this regard, several filament-free switching memristors have been reported based on CMOS-compatible materials [192, 193]. These efforts could facilitate the large-scale industrial integration with filament-free types of memristors. Therefore, to incorporate memrsitive materials and their fabrication methods into the industry, both the selection rule of industry-friendly materials and the diverse technologies to integrate complex and less-compatible materials into CMOS chips need to be further developed, for example back-end-of-line compatible thermal budget control, diffusion barrier materials for high-diffusive ions, and selection/development of cost-effective material. For example, a halide perovskite CsPbBr3-based memristor has been proposed by using a parylene layer as a protecting layer against water and acetone; hence, the photolithography process was successfully performed [194]. For conventional ferroelectric materials such as BaTiO_3_ and Pb[Zr_x_Ti_1−x_]O_3_, there have been several obstacles to the CMOS integration processes, such as high temperature process and the requirement of noble electrodes [195, 196]. Such issues may make ferroelectric hafnia materials more suitable for ferroelectric-based memristors due to its CMOS compatibility [197]. As noted, materials and their combination are all considered for filament-free types of memristors. For example, for ion migration, movable ions such as *V*_O_ are required to be enough within the switching layer to modulate *Φ*_B_ at the interface between switching material and electrode according to the direction of the applied electric field. Conversely, for intercalation, it is required that switching material can function the phase-separation system such that multiphase polarization can be enabled by the (de)intercalated ions driven by the electric field. In addition, the material requirements are also dependent on targeted computing application. For example, conventional vector-matrix multiplication typically requires the static and nonvolatile memory property, whereas reservoir computing favors the dynamic and volatile memory effect. This indicates that the materials and their based junction structures need to be designed and engineered for a desired application.

This Review describes many aspects regarding filament-free types of memristors, their junction structures and switching mechanisms, their use across a range of potential computing applications, and essential discussions. There are convincing reasons to believe that the topics of this Review will provide new opportunities beyond when only filamentary memristors are employed, because the dynamic switching with a variety of internal state variables during the operation can lead to novel functionalities and computation schemes. This, of course, does not imply that filamentary memristors are less important for emerging computing technologies.

## Data Availability

Not applicable.
